# Profiling of extracellular vesicles from primary hepatocytes, organoids, and mash patients identifies cell injury-specific signatures

**DOI:** 10.1038/s41598-026-40490-x

**Published:** 2026-06-03

**Authors:** Aleksandra Leszczynska, Benedikt Kaufmann, Hana Sung, Christian Stoess, Agustina Reca, Andrea Kim, Yeon-Kyung Choi, Chelsea Tran, Sung-Eun Kim, Davide Povero, Bruce Wolfe, Trevor Crafts, Akiko Eguchi, Ariel E. Feldstein

**Affiliations:** 1 Department of Pediatrics, University of California, San Diego, USA; 2 Division of Gastroenterology and Hepatology, Mayo Clinic, Rochester, MN USA; 3 Department of Surgery, Oregon Health and Science University, Portland, USA; 4 Department of Gastroenterology and Hepatology, School of Medicine, Mie University, Mie, Japan; 5 Biobank Center, Mie University Hospital, Mie, Japan; 6 Pfizer, Internal Medicine Research Unit, 1 Portland St, Cambridge, MA 02139 USA

**Keywords:** Extracellular vesicles, Metabolic dysfunction-associated steatotic liver disease (MASLD), Metabolic dysfunction-associated steatohepatitis (MASH), Organoids, Machine learning, Precision medicine, Non-alcoholic steatohepatitis, Translational research, Biomarkers, Diagnostic markers, Predictive markers, Machine learning

## Abstract

Metabolic Dysfunction-Associated Steatohepatitis (MASH) is a severe form of Metabolic Dysfunction-Associated Steatotic Liver Disease (MASLD), traditionally diagnosed via invasive biopsy, underscoring the need for non-invasive alternatives. This study identifies biologically relevant extracellular vesicle (EV) protein signatures associated with MASH using patient serum, primary human hepatocytes (PHH), and human liver organoids (HLO). These complementary models capture distinct aspects of disease progression-circulating EV profiles in patients, hepatic cellular responses in PHH, and multicellular interactions in HLO-providing a comprehensive view of MASH pathophysiology. Using aptamer-based technology, we assayed 6596 proteins from 38 individuals with histological confirmed MASLD and in vitro models. EVs were characterized using nano-flow cytometry, ExoView, and high-resolution microscopy (ONI), with liver-specific markers confirming their origin. Key proteins, including SLC27A5, HP, and CXCL7, were elevated in patient samples, while PHH and HLO models exhibited upregulation of ASGPR1, HP, and CXCL7 under MASH conditions. Proteomic analysis revealed shared pathways across models, with machine learning models achieving AUROC values of 0.97, supporting the diagnostic potential of these protein signatures. This integrative approach advances MASH biomarker discovery by linking localized liver dysfunction with systemic disease mechanisms. These findings highlight clinically relevant EV protein signatures that support the development of non-invasive diagnostics and personalized treatment strategies, including prediction of outcomes for bariatric surgery patients.

**Supplementary Information:**

The online version contains supplementary material available at 10.1038/s41598-026-40490-x.

## Introduction

Metabolic Dysfunction-Associated Steatohepatitis (MASH) is a progressive form of Metabolic Dysfunction-Associated Steatotic Liver Disease (MASLD), characterized by liver cell damage, hepatic inflammation, and varying degrees of fibrosis^1^. Currently, there are limited treatment options for Metabolic Dysfunction-Associated Steatohepatitis (MASH), and diagnosing it typically requires a liver biopsy-an invasive procedure with risks of complications and sampling variability^2–5^. Noninvasive tests (NITs) that can be used for diagnosis, patient segmentation, prognosis, and response to therapy represent a central research priority^4,6^. Additionally, the translational power of various in vitro human-based models for identification and characterization of NITs remains poorly understood^7^. Advancing our understanding of these models could improve MASH diagnosis and treatment, potentially reducing the need for invasive procedures and improving patient outcomes.

While numerous NITs have been proposed, their translational relevance often remains limited by the lack of comprehensive validation across different biological models. Specifically, the use of in vitro human-based models for identifying and characterizing diagnostic biomarkers remains underexplored. To address this gap, our study employs a multi-model strategy, integrating extracellular vesicles (EVs) derived from primary human hepatocytes (PHH), human liver organoids (HLO), and serum samples from MASH patients. Each of these models provides unique insights into MASH pathology: PHH exposed to a defined ‘MASH cocktail’ capture hepatocyte-specific metabolic responses modeling features of MASH, HLO mimics multicellular liver dynamics, and circulating EVs reflect systemic disease processes.

The rationale for utilizing these three models lies in their complementary capabilities. While PHH exposed to a defined ‘MASH cocktail’ enable the exploration of hepatocyte-specific protein signatures modeling features of MASH, HLO provides a more complex, tissue-level perspective that incorporates interactions among liver cell types. Patient serum samples, on the other hand, offer a direct link to systemic manifestations of MASH, facilitating clinical relevance. By integrating these multi-models, we aim to bridge the gap between localized cellular events and systemic disease processes, allowing for cross-validation of protein signatures and enhancing the robustness of biomarker discovery. Current clinical biomarkers for MAFLD/MASH, such as ALT, AST, and imaging-based measures, lack sensitivity and specificity, particularly for early disease detection and progression monitoring. This unmet need underscores the importance of developing more reliable and biologically grounded biomarkers.

Here, we observe that ‘obese’ hepatocyte cultures release unique exosomal proteins, reflected in specific alterations within the organoid EV proteome. By identifying markers that are present in both in vitro models and patient-derived EVs, we add independent evidence supporting the biological relevance of these candidate biomarkers.

The combination of hepatocyte cultures, organoid systems, and patient serum is unique in that it enables mechanistic insights at the cellular level while directly linking them to clinically observable disease states.

These changes are also evident in serum EVs and indicate early-onset MASH, directly influencing metabolic effects. While proteomics measures hundreds of proteins in clinical samples, the complex data processing and interpretation remain a significant challenge^8,9^. Based on exosomal protein sequencing and bioinformatics analysis, we identified specific markers for MASH. Additionally, we applied machine learning-a leading artificial intelligence approach renowned for its ability to automatically analyze complex biological datasets-to further refine and validate these markers^10–12^. This technology offers distinct advantages, especially in interpreting-omics data and developing accurate prediction models^13–15^. Applying machine learning for the analysis of proteomics data to develop biomarkers for MASH is still in its early stages, presenting a major opportunity for groundbreaking research in this field^16,17^. Here, we applied a deep learning approach to EV profiling to explore its potential for distinguishing MASH from healthy controls. This exploratory analysis demonstrated that machine learning can effectively capture EV-based protein patterns associated with disease status. The model’s performance was supported by validation on an independent test set and by consistency with simpler models, such as logistic regression, indicating that the observed separation is not model-specific. Overall, our study highlights that EV profiling captures dynamic and disease-relevant protein signatures that may complement existing diagnostic strategies and enhance understanding of MASH pathology. This not only enhances our understanding of MASH pathology but also has significant potential for clinical applications, including the development of new therapeutic interventions.

## Results

### Enhanced expression and colocalization of hepatocyte-specific and inflammatory markers in MASH-derived EVs

To identify specific proteins that were significantly altered in response to the distinct cellular environments of MASH, we initially focused on the impact of EVs derived from supernatants of MASH-induced primary human hepatocytes (PHH) and human liver organoids (HLO). Alongside, PHH and HLO of healthy subjects were also investigated in the same manner. Circulating EVs from MASH patients and healthy subjects were also studied (Table 1). This multifaceted experimental setup was designed to discern the interplay between cellular environments and the resulting composition and content of circulating EVs.

**Table 1:** Baseline characteristics of the study population.

		Total (n = 17)
Demographics	Age	42(28–63)
	Female	62%(11)
	Body mass index (kg/m^2^)	47(37–66)
Histology	Fibrosis score 0–1	33%(6)
	Fibrosis score 2–3	39%(7)
	Healthy	22%(4)

All data are presented as the median or percentage(n).

**Fig. 1:**
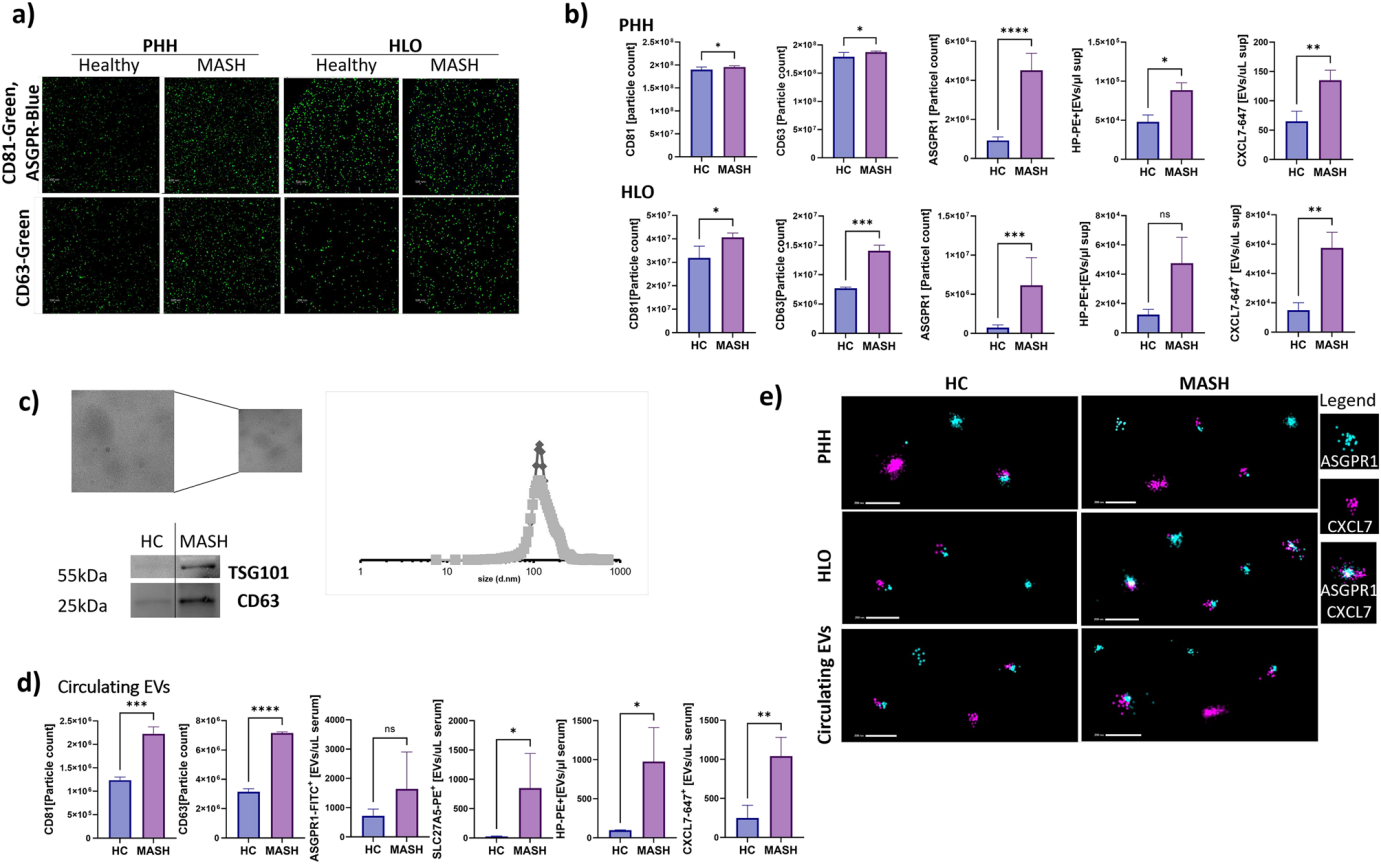
Identification and characterization of circulating extracellular vesicles (EVs) in the supernatants from human hepatocytes, organoids, or serum from MASH patients were carried out as follows: (**A**, **B**) NanoView images and quantification of CD81, CD63 and ASGPR1 and NanoFCM analysis of HP and CXCL7 was employed for the identification of supernatant EVs and their characterization in MASH treated PHH or HLO samples. Scale bars 500 nm. (**C**) Cryo-electron microscopy images were captured to represent circulating EVs from MASH patients (representative images). The size of circulating EVs derived from MASH was determined using nanoparticle tracking analysis (DLS), resulting in a size of approximately 140 nm. Western blot analysis of EV marker shows CD63 and TSg101 in Healthy control and MASH derived circulating EV protein lysates. (**D**) NanoView of CD81, CD63 of EVs detected in healthy controls (n = 4) vs MASH patients (n = 8). NanoFCM of circulating EVs positive for known hepatocyte markers ASGPR1-488, SLC27A5-PE, or novel markers CXCL7-647, HP-PE. (**E**) ONI (high resolution microscopy) of EVs derived from HLO, PHH and circulating EVs with colocalization for CXCL7-647(pink) and ASGPR-488 (cyan blue). Scale bars, 200 nm. All data presented as mean ± SD (n = 4). *P* values calculated by two-sided unpaired *t*-test.

The established EV markers CD63 and CD81^18^ were significantly increased in the MASH groups (PHH, HLO cultures as well as circulating EVs) compared to their respective healthy controls as assessed by NanoView (Fig. 1b, d). Hepatocyte-specific markers ASGPR, haptoglobin (HP), and C-X-C motif ligand 7 (CXCL7) expressing EVs were also significantly increased (Fig. 1b, d).

Furthermore, we extended this analysis to investigate the differences between healthy control subjects and individuals diagnosed with MASH (Fig. 1c, d).

Based on our previous reports that show an increase of circulating EVs in cohorts spanning from pre-cirrhotic to cirrhotic MASH^19^, we conducted a thorough analysis of individuals with MASH (F0, F1, F2, F3, n = 13) versus healthy controls (n = 4) as detailed in Table 1.

Circulating EVs from MASH patients were detected in serum using Cryo-EM microscopy. The median EV size was around 150–200 nm (Fig. 1c). These EVs were positive for EV markers, such as CD63 and TSG101, an integral component of the ESCRT-1 protein complex for the synthesis and secretion of EVs (Fig. 1c). Moreover, these markers were also significantly elevated in circulating EVs from MASH patients (Fig. 1d). Additionally, in circulating EVs the bile specific-coenzyme A synthetase (SLC27A5) was shown to have significantly higher expression (Fig. 1d). SLC27A5 expression is upregulated in fat-laden hepatocytes as this enzyme plays an important role in facilitating the uptake, elongation, and synthesis of fatty acids^20^. Taken together, these findings suggest a possible link between fatty acid metabolism and alterations in insulin signaling pathways, which may contribute to the development of insulin resistance.

High resolution microscopy (ONI) showed colocalization of the ASGPR1 and CXCL7 in EVs from HLO and PHH, as well as circulating EVs (Fig. 1e). These results suggest that hepatocytes undergoing stress may be a source of CXCL7 that drives inflammation^21–23^. Furthermore, circulating EVs from MASH patients exhibited staining patterns of CXCL7 similar to those of ASGPR1-positive vesicles, suggesting a potential association of CXCL7 with liver-derived EVs. However, co-expression of both markers may not consistently be observed. This finding is consistent with the possibility that liver injury in MASH may be accompanied by increased secretion of EVs, although further studies stratified by disease stage are needed to directly establish this association.

### Proteomic profiling of EVs from PHH and HLO Reveals Disease-Specific Protein Signatures in MASH conditions

**Fig. 2:**
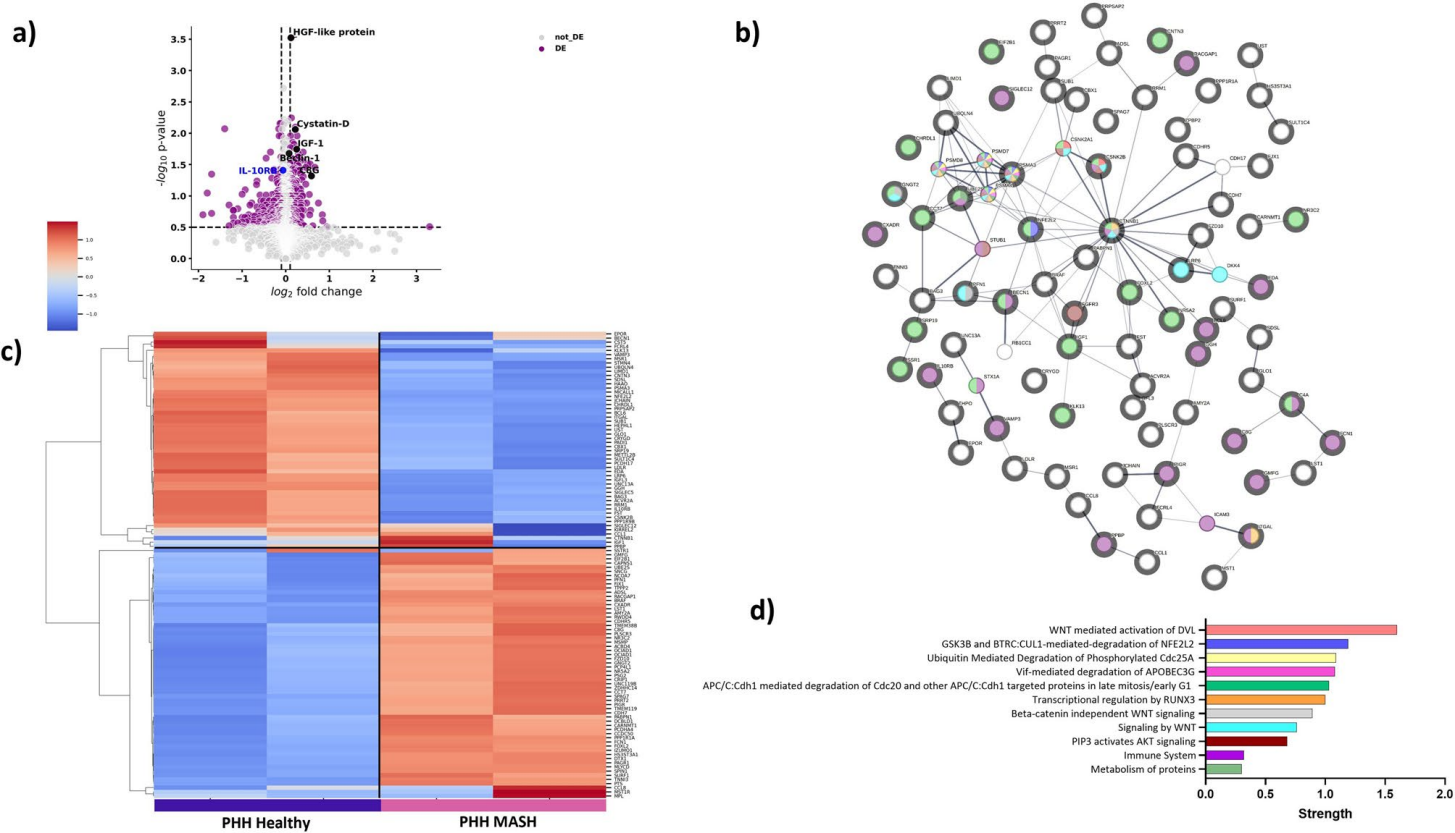

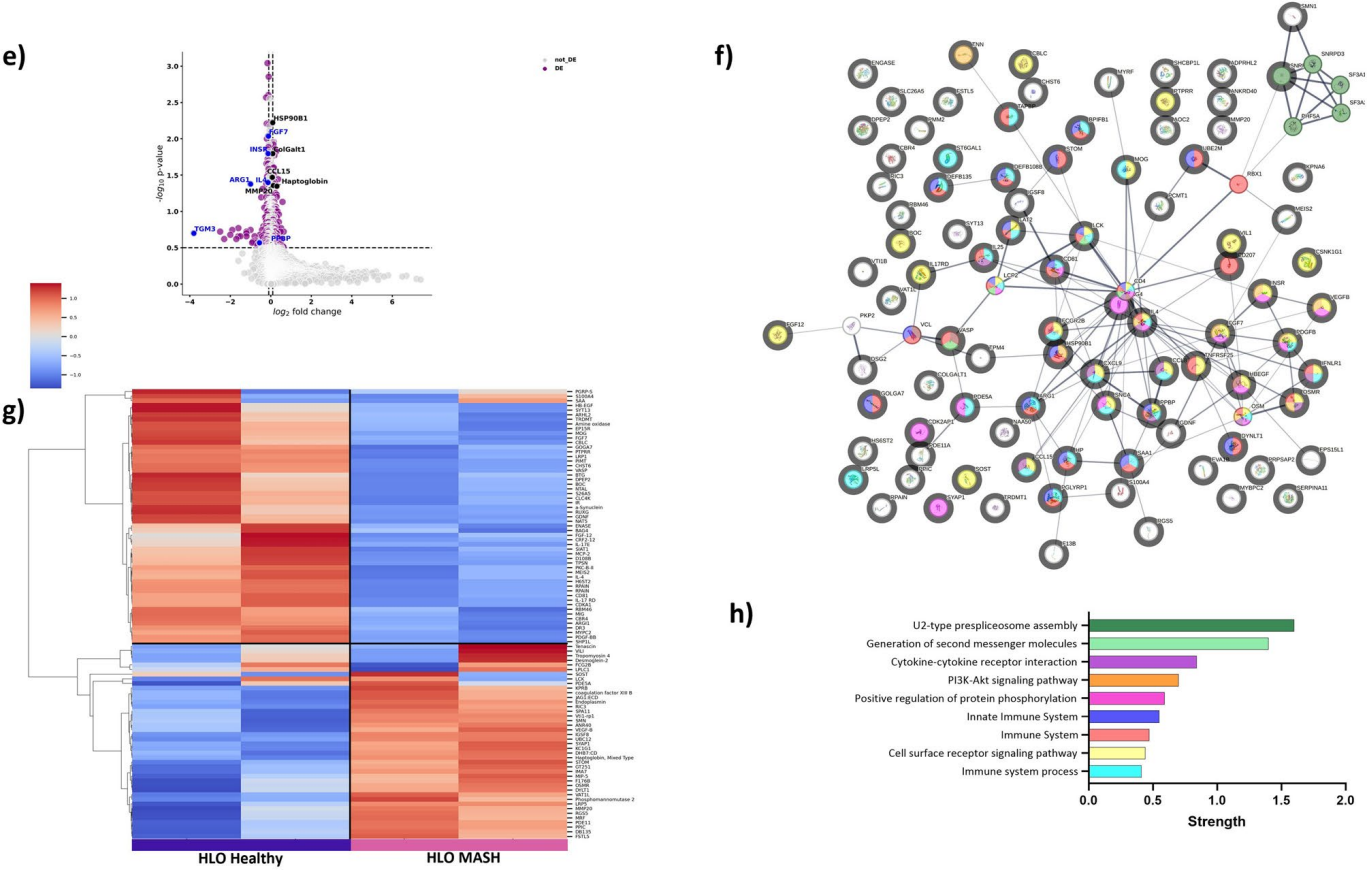
Proteomics analysis of EVs from PHH and HLO supernatants. Volcano plot illustrating significantly abundant EV- proteins in PHH MASH vs PHH Healthy controls, (**A**) and HLO MASH vs HLO Healthy controls (**E**). The –log10 (p-value) is plotted against the log2 fold-change. Proteomics analysis of EVs from PHH, (**B**) and HLO (**F**). Unsupervised hierarchical clustering analysis of top 100 differentially expressed EV-proteins in PHH Healthy (n = 6) vs PHH MASH (n = 6) pulled samples, (**C**) and HLO Healthy (n = 4) vs HLO MASH (n = 4) pulled samples (**G**). Top 100 proteins were selected based on the p-value of 0.05 from the MASH vs Healthy controls comparisons. Mean and SD of all groups were used to normalize the expression value. Reactome Pathway enrichment analysis of total differentially expressed EV-proteins of PHH, (**D**) and HLO (**H**). Key pathways are marked by signature protein involvement and highlight their utmost importance in the context of MASH. The threshold was set at a p-value greater than 0.05. Data points situated above the horizontal line parallel to the non-axial axis indicate EV-protein p-values exceeding 0.05. Notably, when points are positioned to the left of the vertical line that intersects the non-axial axis, this signifies down-regulated EV proteins. Conversely, if the points are located to the right of this vertical line, they denoted as up-regulated EV proteins.

To establish whether EVs released by PHH and HLO offer a translational platform capable of identifying and quantifying specific EV subpopulations and their clinical implications, we examined the protein composition of EVs from both healthy and MASH-induced conditions (as described in methods section). We conducted an in-depth analysis of EVs derived from PHH (Fig. 2a–d) and HLO (Fig. 2e–h), utilizing an unsupervised and comprehensive proteomics approach. EV protein lysates from both groups were validated for their proteomic signature using the SomaScan Assay^24^. To visually represent the complete set of statistically significant EV proteins, we generated volcano plots. These plots illustrated the relationship between the -log10(p-value) and the log2 fold-change in two distinct comparisons: PHH vs. PHH with MASH-like induced condition (Fig. 2a) and HLO vs HLO with MASH-like induced condition (Fig. 2e). 116 proteins (68 up-regulated and 48 down-regulated) were identified as differentially expressed in MASH PHH EVs (as compared to untreated healthy PHH). Similarly, in HLO EVs we identified 97 (37 up-regulated and 60 down-regulated) differentially regulated proteins (as compared to untreated healthy HLO). An unsupervised hierarchical clustering analysis of the top 100 proteins identified distinct expression patterns particularly between healthy controls and MASH induced cultures, suggesting that protein composition of EVs is strongly associated with the cell type by which it is secreted (Fig. 2c, g). Furthermore, we identified a distinct PHH vs. HLO EVs MASH protein profile. The top upregulated proteins in PHH MASH EVs include Cystatin-D, Macrophage stimulating 1 (MST1), Insulin-like growth factor 1 (IGF1), while Interleukin 10 receptor subunit β (IL10RB) was downregulated. These proteins are involved in inflammatory and immune responses, autophagy, cell cycle control, cell senescence, cell motility, endocytosis, insulin sensitivity, tissue development, and homeostasis^25–28^ (Fig. 2b, d). Similarly, the top upregulated proteins in HLO MASH EVs are Haptoglobin (HP), Matrix metalloproteinase 20 (MMP20), Collagen galactosyltransferase 1 (ColGalT1), Heat shock protein 90 beta family member 1 (HSP90B1), C–C motif chemokine ligand 16 (CCL16), and downregulated proteins Arginase 1 (ARG1), Insulin Receptor 1 (INSR), Fibroblast Growth Factor 7 (FGF7), and Transglutaminase 4 (TGM4)^20,29,30^ (Fig. 2f, h). The differential expression of insulin-related proteins in PHH (Primary Human Hepatocytes) and HLO (Hepatic Liver Organoids) EVs underscores the complexity of insulin sensitivity and resistance in MASH. The upregulation of IGF1 in PHH MASH EVs suggests a potential compensatory mechanism aimed at enhancing insulin sensitivity, as IGF1 plays a key role in promoting glucose uptake and supporting insulin signaling pathways^31,32^. This upregulation might reflect an early attempt by hepatocytes to counteract insulin resistance and maintain metabolic balance. The downregulation of INSR in HLO MASH EVs significantly disrupts insulin signaling, contributing to insulin resistance and highlighting the role of Kupffer cells and stellate cell activation in exacerbating the MASH phenotype. This decreased expression of INSR suggests reduced cellular responsiveness to insulin, worsening metabolic dysfunction. The contrast between IGF1 upregulation in PHH and INSR downregulation in HLO underscores the different stages and mechanisms of insulin resistance across cellular models of MASH, offering insights into disease progression and potential therapeutic targets.

Our proteomic data suggests that EVs derived from MASH-induced conditions are enriched with distinct proteins, which provide multi-protein signature. This observation potentially unveils a promising avenue for employing these signature proteins as valuable biomarkers in the further assessments and diagnosis of MASH.

### Differential protein expression in circulating EVs reflects early stages of liver fibrosis in MASH patients

**Fig. 3:**
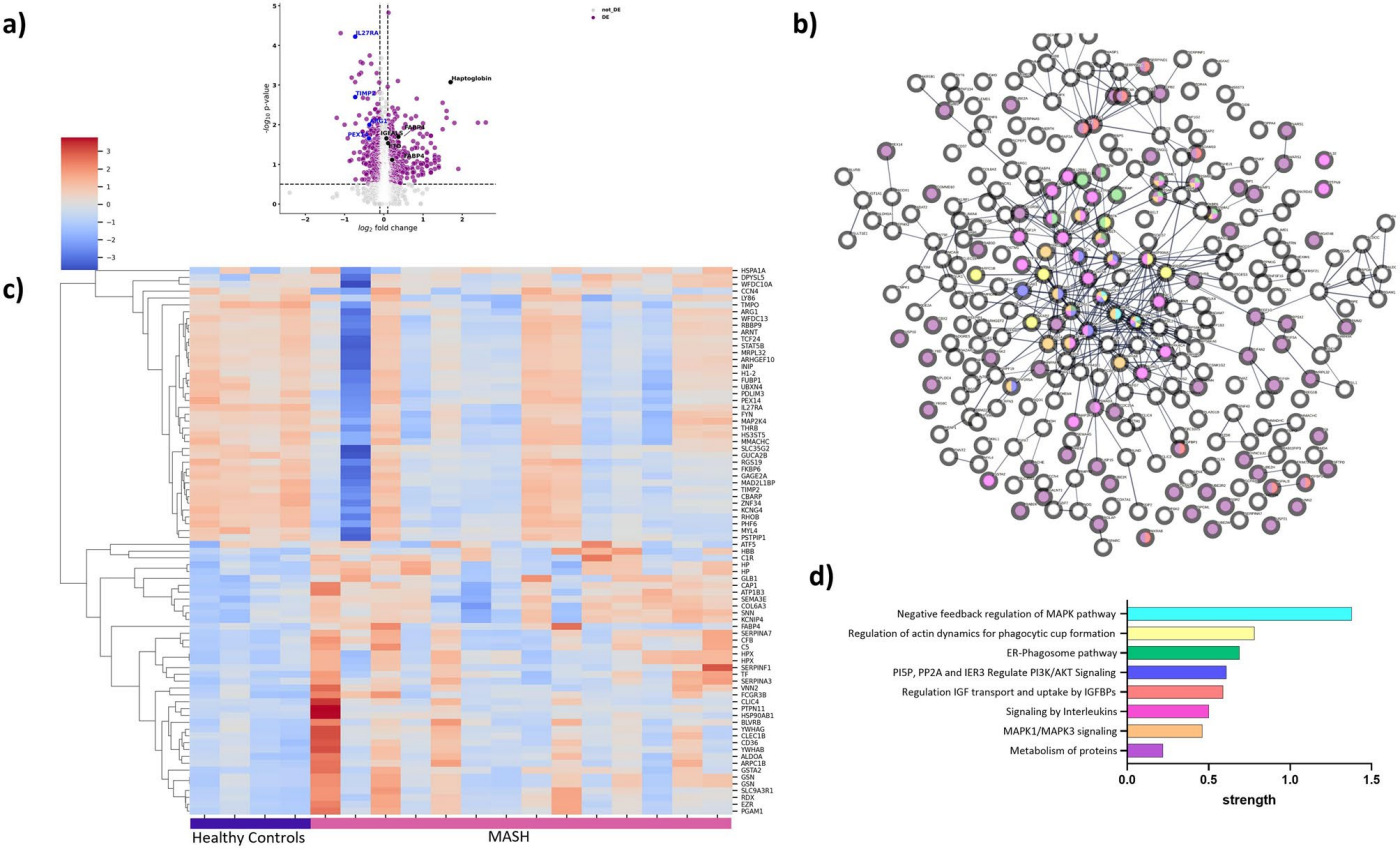
Proteomics analysis of circulating EVs isolated from MASH (total) patients. (**A**) Volcano plot illustrating significantly abundant EV- proteins in total MASH patient’s vs Healthy controls. The –log10 (p-value) is plotted against the log2 fold-change. (**B**) Proteomics analysis of EVs from total MASH patients. (**C**) Unsupervised hierarchical clustering analysis of top 100 differentially expressed EV-proteins in Healthy controls (n = 4) vs total MASH patients (n = 14). Top 100 proteins were selected based on the p-value of 0.05 from the MASH patient’s vs Healthy controls comparisons. Mean and SD of all groups were used to normalize the expression value. (**D**) Reactome Pathway enrichment analysis of total differentially expressed EV-proteins. Key pathways are marked by signature protein involvement and highlight their utmost importance in the context of MASH.

The findings of the differentially expressed proteins in EVs from PHH and HLO with MASH led us to further investigate whether EV protein composition was significantly different in MASH patients with various stages of fibrosis (F0, F1, F2, F3, showed in Table 1). Indeed, previous studies have demonstrated that the protein composition of circulating EVs reflects the health status of the cells and is dynamically reflective of specific diseases, disease stages, or responses to therapeutic interventions^20,33^. Our study uncovered previously unrecognized proteomic profiles associated with various stages of liver fibrosis in MASH. Top upregulated proteins in EVs with MASH (HP, Fatty acid-binding protein 4 -FABP4, Insulin-like growth factor binding protein acid labile subunit -IGFALS, Ferritin, and fat mass and obesity-associated protein -FTO) are involved in cell growth, differentiation, metabolism regulating lipid storage and insulin sensitivity, associated with obesity and metabolism dysregulation^34,35^ (Fig. 3a). Among these, HP emerged as a unique marker for MASH, detected consistently across HLO, and patient EVs. The downregulated proteins include ARG1, tissue inhibitor of metalloproteinases 2 -TIMP2, Interleukin 27 receptor subunit alpha -IL27RA, and peroxisomal biogenesis factor 14 -PEX14. And each is involved in distinct processes like: ARG1 is linked to arginine metabolism^36^, TIMP2 regulates matrix metalloproteinases (MMPs) associated with immune responses and inflammation^37^, IL27RA plays a pivotal role in peroxisomal function^38^, and PEX14 is involved in regulating fatty acid peroxidation^39^ (Fig. 3a, b). The findings from our proteomic analysis strongly indicate that circulating EVs present a distinct array of proteins.

Out of the unique analytes that are in the SomaScan Assay, 423 proteins (226 were up-regulated and 197 were down-regulated) were differentially expressed in EVs from MASH cohort compared to healthy control. The top 100 differentially expressed proteins, visualized through unsupervised hierarchical clustering (Fig. 3c), highlighted pathways central to disease progression. These included core protein–protein interactions such as the MAPK pathway, AKT signaling, IGF transport, and interleukin signaling. Additionally, pathways regulating actin dynamics, ER-phagosome interactions, and lipid metabolism were enriched in the dataset (Fig. 3d).

By analyzing proteins across PHH, HLO, and patient EVs, we demonstrated that these models inform one another and collectively capture both localized liver dysfunction and systemic disease processes. Several pathways, such as the MAPK and AKT signaling pathways, were consistently implicated across all models, underscoring their importance to MASH pathology. This integration allows for the identification of multiprotein-based signatures that are specific to early disease stages and the progression of fibrosis.

Overall, these findings establish a foundation for developing novel noninvasive diagnostic tools. By leveraging the multi-model approach, we aim to generate robust, clinically relevant biomarkers that reflect the complex pathophysiology of MASH and its progression.

## Deep learning model accurately predicts MASH patient outcomes: validating proteomic data from PHH, HLO, and circulating EVs for reproducible diagnostic signatures

The rapid advancements in deep learning tools have significantly improved our capabilities in biomarkers development. We leverage the distinctive significant protein signatures identified in PHH, HLO and circulating EVs to develop innovative MASH diagnostic approach. We integrated panels representing steatosis, insulin signaling and sensitivity, inflammation, hepatocyte ballooning, and fibrosis into our analysis. Machine learning was used to develop a model for predicting clinical status in this study. Using the Ridge regression algorithm^40^ (Supplementary data for more details), we selected 19 essential protein markers (Table 2). The robustness of these signatures was further supported by orthogonal models, including logistic regression and k-NN classifiers, which showed consistent separation of MASH from controls (Supplementary Sects.  1.2–1.4). Subsequently, we performed a comparative analysis using Ridge feature coefficients to assess the importance of each feature in predicting the target variable (Supplementary Fig. 1).

**Table 2:** Input disease biomarkers for machine learning.

Input disease biomarkers				
HP	SERPINA3	PTPA	TIMP2	PPBP
HPX	GSTA2	FTH1	FYN	S100A4
SERPINA7	CLEC1B	ARG1	IL27RA	SAA1
ANKRD	FABP4	IGFBP1	OSMR	

List of the future selection inputs (potential biomarkers) for disease biomarkers.

**Fig. 4:**
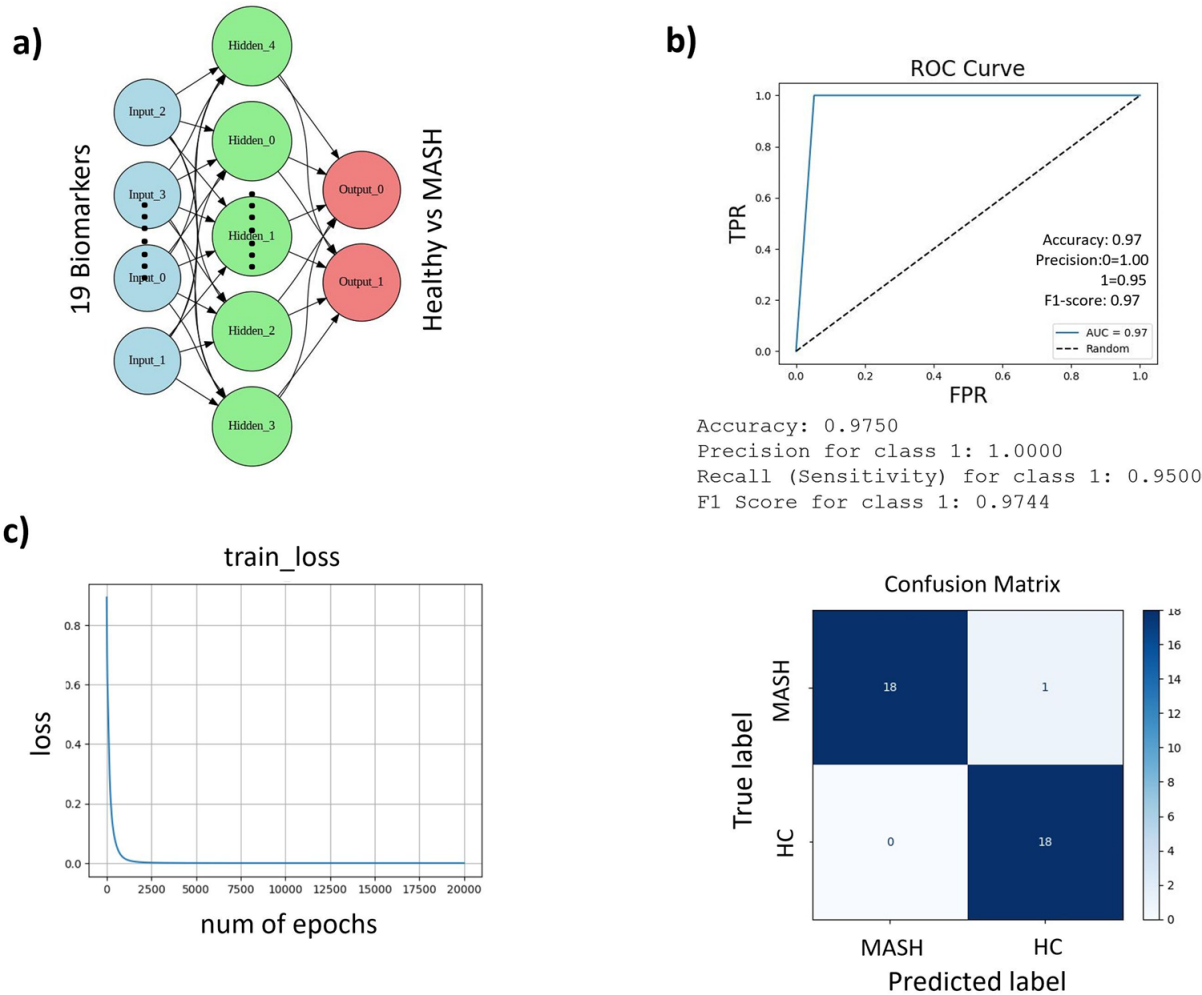
Unbiased estimation of Circulating EVs predictive ability to detect MASH. (**A**) Representative architecture of Neural Network (ANN) used. Each input node represents a biomarker, fully connected network with the hidden layer. Each arrow represents multiplication of the input or activation by a weight. A convolutional layer, Input 1- 19 are the inputs to the layers and 1 -7 hidden layers with the 2 binary outputs layers 1, and 0. (**B**) ROC curves for the validation cohort predicting the presence of MASH. The ROC curves demonstrate the robustness of the model, with an AUC of 0.97 for the test data. The dotted diagonal line represents random performance. The figure includes accuracy, sensitivity, and specificity metrics for MASH vs HC. It also includes a confusion matrix for the Bariatric surgery MASH cohort, showing true and predicted label. (**C**) Training curves of the ANN show the decrease of the mean squared error (MSE) loss during training. The MSE quantifies the deviation of patient chart MASH score ground truth and ANN predictions. Receiver operating characteristic (ROC) curve and the confusion matrix for the Neural Network Classifier model to classify 18 positive (MASH) vs. 19 negative (Healthy Control) individuals (b). AUC, area under the curve; FPR, false-positive rate; TPR, true-positive rate; NN, neural network.

Next, we used the Keras framework to implement and train a neural network model with 19 selected features. Our primary objective was to perform binary classification, accurately predicting whether new objects belong to the Healthy class (0) or the MASH category (1). The neural network architecture was designed with 19 input biomarkers (Table 2), 7 neurons in the hidden layer, and 2 neurons in the output layer (Fig. 4a). Using python, we leveraged Keras (version 2.14.0) integrated with TensorFlow to facilitate model development within the Google Cloud environment. The number of neurons in the hidden layer was treated as a hyper-parameter. Laser features used in the k-NN method or logistic regression model served as independent analysis of deep learning model (supplementary materials).

The Sequential model was instantiated, followed by the addition of layers, specifying the activation functions, and model compilation with categorical cross-entropy loss and ADAM (Adaptive Moment Estimation) optimizer.

Training the model involved 20,000 epochs on the provided training features and labels, with the loss function converging to zero, indicating successful model convergence (Fig. 4c).

Further assessment of the model’s performance involved evaluating its predictions against known outcomes in the validation Bariatric Surgery MASH cohort (see Supplementary methods). Baseline demographic features are depicted in Table 3.

**Table 3:** Baseline characteristics of the test population.

		Total(n = 21)
Demographics	Age	52(26–70)
	Female	81%(17)
	Body mass index (kg/m^2^)	53(36–65)
Histology	Fibrosis score 0–1	38.1%(8)
	Fibrosis score 2–3	68.9%(13)

After carefully selecting features and hyperparameters (Fig. 4a, Supplementary data), we tested our model using accuracy scores, confusion matrices, and receiver operating characteristic (ROC) curves (Fig. 4b). The confusion matrix assesses a classifier at the fixed threshold, while ROC evaluates that classifier across all possible thresholds. The area under the ROC curve (AUC) serves as a performance metric across the classification threshold. A higher true-positive percentage and a lower false-positive percentage will produce better AUC results. The model, validated on the test set (Bariatric Surgery cohort), achieved an AUROC of 0.972 (95% CI: 0.90–1.000), with a sensitivity of 0.95, and specificity of 0.97 (Fig. 4b). Utilizing this panel enhances the probability of accurately differentiating between a MASH patient and a healthy individual to 97% by detecting levels of the relevant biomarkers in circulating extracellular vesicles (EVs). The confusion matrix further demonstrated the model’s overall accuracy, showing a 97.5% accuracy score in predicting test cases, with only one misclassified instance, which aligns closely with expectations (Fig. 4b, Confusion Matrix). This evaluation shows the confidence in the neural network’s ability to correctly classify disease and non-disease states in the given dataset. The model’s overall accuracy, precision, recall, and F1 score (Supplementary materials 1.2), coupled with the details from the confusion matrix, collectively affirm the neural network’s adeptness in accurately classifying disease and non-disease states within the Bariatric Surgery MASH cohort, while also providing valuable insights for potential refinements.

### Independent validation of proteomic signatures in a human cohort that underwent bariatric surgery confirms distinct EV protein profiles

**Fig. 5:**
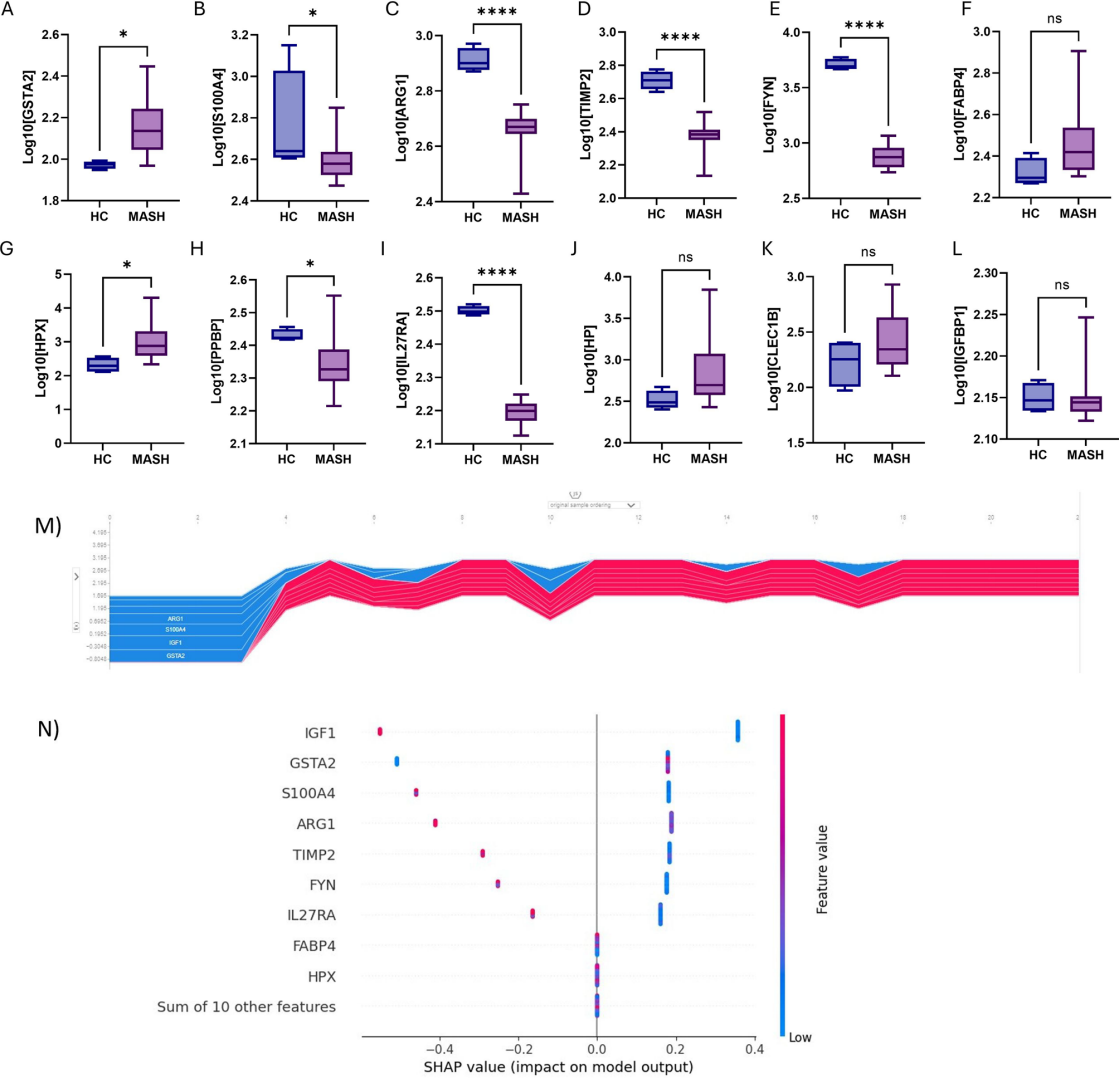
Comparison of different levels of EVs proteins in a validation cohort of patients with bariatric MASH, and Healthy Controls. Boxplots of comparison of top highest expressed circulating EV proteins isolated from subjects. Data are reported as log10(RFU) for the EVs, (**A**) GSTA2, (**B**) S100A4, (**C**) ARG1, (**D**) TIMP2, (**E**) FYN, (**F**) FABP4, (**G**) HPX, (**H**) PPBP(NAP-2), (**I**) IL27RA, (**J**) HP, (**K**) CLEC1B, (**L**) IGFBP1. A regular Student *t* test (comparing means of raw and log10 values) was used to generate the *P* value reported in the boxplots. SHAP values and feature interaction scores in XGBClassifier-based prediction. (**M**) SHAP Stacked Force plot (interactive) with passed all 23 observations into the Force plot function displaying values distribution across the dataset. (**N**) The top 9 most important features for the prediction of MASH recurrence (ranked from most to least important). The distribution of the impacts of each feature on the model output. The colors represent the feature values: red for larger values and blue for smaller values. Abbreviations: IGF1: Insulin-like Growth Factor 1; GSTA2: Glutathione S-Transferase Alpha 2; S100A4: S100 Calcium Binding Protein A4; ARG1: Arginase 1; TIMP2: Tissue Inhibitor of Metalloproteinases 2; FYN: Tyrosine Protein Kinase; IL27RA: Interleukin 27 Receptor Subunit Alpha; FABP4: Fatty Acid Binding Protein 4; HPX: Hemopexin.

Additionally, we conducted an independent validation study wherein we replicated the SomaScan protein Assay analysis on a Bariatric Surgery (BS) MASH patient cohort. The expression levels of most relevant circulating EV proteins in total MASH patients (HP, HXP, IL27RA, C-type lectin domain family 1 member B -CLEC1B, ARG1, insulin-like growth factor-binding protein 1 -IGFBP1, pro-platelet basic protein -PPBP, fatty acid-binding protein 4 -FABP4, glutathione S-transferase A 2 -GSTA2,) were compared to Bariatric Surgery patient cohort versus Healthy controls. Interestingly, our analysis reveals a distinct separation in the distribution patterns of all twelve proteins (Fig. 5a-l). For instance, HP exhibited a log10-transformed median expression of 2.7 in the Bariatric Surgery MASH group compared to 2.49 in Healthy controls (Fig. 5j), while HPX showed a log10-transformed median expression of 3.31 in the Bariatric Surgery MASH group versus 2.79 in Healthy controls (Fig. 5g). To enhance the clarity of our results, we utilized SHAP (Shapley Additive Explanations) to interpret the predictions^41,42^. SHAP provides a score for each input variable, reflecting its contribution to the XGBClassifier model’s prediction. In our visualizations, SHAP values are plotted on the y-axis, with different features grouped accordingly. The color of the points indicates the magnitude of the feature values, with higher values shown in red. The x-axis denotes the SHAP values, which illustrate each feature’s influence on the prediction. Higher values of certain markers, such as TIMP2, S1004, and ARG1, tend to negatively affect the model’s output, as evidenced by their negative SHAP values. This visualization highlights the significance of the relationships between features and model outcomes (Fig. 5m, n).

This comprehensive analysis of our research findings substantiates the understanding that utilizing distinctive protein based EVs signatures could serve as a valuable diagnostic. It underscores the potential clinical utility of leveraging EV-associated protein profiles in the accurate identification and diagnosis of MASH patients.

## Discussion

In this study, we utilized diverse sources of extracellular vesicles (EVs) to investigate protein alterations in serum from MASH patients and healthy controls. Our aim was to identify circulating EVs with potential diagnostic value. Specifically, we emphasized the importance and effectiveness of in-depth proteomic analysis using our neural network model for protein-based liquid biopsies. Given that MASH with fibrosis is a reversible condition following lifestyle and pharmacologic interventions, there is a growing need for noninvasive monitoring. Increasing evidence indicates that leveraging the protein content of EVs as a liquid biopsy assay offers a promising strategy for early detection in various human diseases^43^. This is primarily due to their small size, resistance to nuclease-mediated degradation, and their abundant, and tissue-specific presence in different bodily fluids. Nevertheless, there is ongoing debate regarding whether protein liquid biopsies, released from multiple cell sources, offer sufficient diagnostic specificity for detecting a specific disease condition as well as provide information regarding severity of the disease^44^. Recently, there has been a growing interest in investigating MASH-derived EVs and their protein cargo^19,45–47^. This exploration holds the potential to provide additional measures of specificity needed to enhance diagnostic accuracy in patients with MASH.

Unlike traditional bulk serum proteomics, which captures a heterogeneous mixture of proteins largely influenced by abundant serum proteins, our EV multi model-based proteomics offers a more refined and cell-source–specific readout. By enriching for MASH vesicle-associated proteins, this approach can reduce background noise and highlight disease-relevant molecular signatures with greater specificity.

Considering the tight association between MASH and its proteomic profile, our work emphasized the predictive value of EVs proteome in MASH. The hypothesis of this study is that a combination of EVs derived from culture supernatants from human-based in vitro models and circulating EVs from patients with MASH enables a measurement of hundreds of proteins, thus could uncover as many potential biomarkers as possible^48^.

By integrating human hepatocytes, liver organoids, and patient-derived EVs, our study establishes a translational framework that bridges experimental models with clinical samples. EVs were analyzed from hepatocyte and organoid cultures with a MASH-induced phenotype, as well as from blood samples of patients with varying degrees of fibrosis and MASH and from non-disease controls. This combined strategy enhances biomarker discovery by ensuring biological relevance across systems and supports the identification and validation of a proteomic signature for the noninvasive detection of MASH. Previous studies have used a machine learning approach to discern a panel of circulating proteins capable of effectively distinguishing between healthy individuals and those afflicted with MASH, based on their serum proteomic profiles^30,49–52^. However, this study did not look at specific fractions within the serum and liver specific signals were not dissected^30^. Our study focused on analyzing protein content of isolated EV in early stages of MASH specimens for the discovery of candidate protein biomarkers, as well as train, test and validated performance of the candidate biomarkers in an independent patient cohort study. In our previous research, we developed experimental EV signatures that could distinguish between healthy controls and patients with pre-cirrhotic MASH or cirrhotic MASH^19^. The AUROC values of 0.77 (95% CI: 0.5635–0.9103) for advanced MASH versus healthy controls and 0.8 (95% CI: 0.5634–0.9427) for pre-cirrhotic versus cirrhotic patients, obtained from the top 10 kTSP-selected EV protein pairs, signify moderate and good accuracy, respectively, in distinguishing between patient groups^19^.

To better understand the functional relevance of the biomarker candidates we designed our experiments with the implementation of dissecting the EV protein content. In our comprehensive approach utilizing a proteomic array with over 7,000 unique analytes, we identified a considerable number of differentially expressed proteins in circulating EVs from both healthy individuals and MASH patients, as well as in cell culture supernatants. This unique cargo in circulating EVs specific to MASH disease, when compared with EVs from hepatocytes and organoids, suggests that MASH-specific protein signatures could serve as a critical element in replicating MASH in-vitro. This dual functionality could pave the way for early diagnosis of MASH and advancing treatment development. The data was further filtered to retain 423 target proteins based on log2-fold change > 0.5 and p-value < 0.05 that were differentially expressed between MASH patients and heathy subjects. Many of the proteins identified showed a strong functional relevance and could be linked to key processes involved in MASH pathogenesis including lipid and glucose metabolism^53^ and signaling by interleukins^54^ annotated in the STRING analysis (version 12.0). Furthermore, functional annotation based on REACTOME pathway analysis showed these target proteins were enriched in phagosome pathway, MAPK signaling and IGF transport and uptake by IGFBPs.

Our approach relies on partial sequencing of the proteome, which involves mapping the obtained sequences to predefined sets of candidate proteins. This method not only enhances our understanding of EV-derived proteins but also opens new possibilities for proteomic studies in clinical research. Looking ahead, we anticipate that further developments in this platform will continue to enhance the accessibility and depth of proteomic studies, leading to more significant discoveries in the field of clinical research.

Liver biopsy-based disease grading and staging remains the gold standard for diagnosis of MASH and therefore we compared the performance of combined protein signature with respect to standard practice^55–58^. This helped develop a clinically feasible assay that includes only the panel of markers required for maintaining overall diagnostic performance. In recent years, machine learning algorithms have been increasingly employed to uncover associations between -omics data and disease status and to create predictive models^59,60^. Accordingly, we leveraged machine learning algorithms in our study, demonstrating their capability in three key areas.

First, to avoid overfitting, we implemented the Ridge regression algorithm to reduce the impact of less important features among differentially expressed protein groups in our deep learning model. Second, compared to previous biomarker discovery studies that utilized logistic regression and random forest algorithms, we chose a neural network to train the model, therefore achieving higher robustness^61,62^. Third, our algorithm successfully revealed the predictive potential of proteins that traditional analytical methods often overlook or miss. For instance, selected proteins exhibited strong predictive power for the presence of early MASH versus healthy control samples. Our 19-biomarker panel demonstrated a 97.5% accuracy in the confusion matrix for diagnosing early MASH. Importantly, we consider the neural network results as exploratory rather than definitive. The neural network model was supported by validation in an independent patient cohort and consistency with simpler models such as logistic regression and k-nearest neighbors. Together, these orthogonal approaches strengthen confidence that the observed biomarker signatures reflect biologically relevant signals.

The translational feasibility of this protein panel may rely on the adaptation of emerging high-throughput proteomic platforms that could help bridge the gap between discovery and clinical application.

In summary, the strengths of our study include the use of three types of EV cohorts and highly sensitive targeted protein analysis of MASH and healthy controls. This approach illustrates the proteomic reprogramming landscape of MASH and provides a valuable resource that expands our knowledge of the disease. Furthermore, the application of machine learning and proteomics presents significant advantages, complementing a range of studies on MASH characterization and precision medicine. Additionally, our model was constructed based on a simple set of proteins, facilitating replication, optimization, and clinical application.

The top biomarkers (Haptoglobin, Hemopexin, IL27RA, CLEC1B, Arginase1, IGFBP1, PPBP, FABP4, and GSTA2) are proteins involved in various key processes such as metabolic dysregulation, inflammation, and fibrosis^63–65^. For instance, FABP4 (Fatty Acid-Binding Protein 4) and IGFBP1 (Insulin-like Growth Factor Binding Protein 1) are crucial in regulating lipid metabolism and insulin sensitivity. These findings highlight the potential roles of these biomarkers not only as indicators but also in the underlying pathophysiology of MASH. Insulin signaling and resistance are central to the progression of metabolic syndrome, and several of these markers are integral to our machine learning model. This underscores the significant impact of type 2 diabetes in the context of MASH, emphasizing its importance in understanding and identifying the disease^66–68^. Building on this, our study provides compelling evidence directing further investigation into the potential role of the CXCL7-CCR2 axis in MASH development^69–71^. Binding CXCL7 can affect proliferation and migration, and it is involved in DNA synthesis, glycolysis, mitosis, intracellular cAMP accumulation, prostaglandin E2 secretion, and hyaluronan and fibrinogen activation ^72^. This suggests that EVs CXCL7 could potentially contribute to the activation of the levels of HA (Hyaluronic Acid) potentially influencing the development and progression of MASH^65,73^. Furthermore, the persistent presence of CXCL7 in circulating EVs suggests its potential as a reliable biomarker for assessing liver health and pathology in MASH patients, providing valuable insights for diagnosis and monitoring. Another marker that is present throughout the EVs analysis is haptoglobin (HP)^29,74^. HP is predominantly synthesized by hepatocytes, and its levels are increased once infection, inflammation, or injury occurs^75^. These findings may suggest a secretion of the HP may involve EVs. The marker’s involvement in inflammatory response suggests it could be a promising biomarker for progression of MASH. Previous studies have suggested that fucosylated haptoglobin could serve as a potential marker distinguishing MASH from simple steatosis^76,77^. Notably, the characteristic feature of typical pathological MASH involves ballooning hepatocytes, wherein the polarized secretion of proteins is disrupted due to the destruction of the cytoskeleton of these cells. This disruption in cellular architecture may contribute to the observed changes in haptoglobin expression levels, providing insights into the distinctive features associated with MASH.

In this study, the EV-based protein signatures demonstrated significant diagnostic accuracy, indicating their potential to complement existing diagnostic approaches for MASH.

The potential limitations of this study include the sample size of the two patient cohorts used in this study, although the cohorts included span across distinct stages of fibrosis the numbers were relatively small. Further prospective studies with larger cohorts will be important to translate these findings into clinical settings. Additionally, enhanced access to patient clinical data would prove advantageous in executing more advanced predictive models, harmoniously integrating the utilization of deep learning tools with clinical evaluation.

In summary, to the best of our knowledge, this is the first multi-model study to report the performance of EVs isolated from human hepatocytes, organoids, and circulating EV from serum in the diagnosis of MASH. The utilization of systematic and comprehensive biomarker discovery, coupled with clinical samples, offers novel evidence for the relevance of EVs and their protein cargo for the development of MASH biomarkers. This work also represents an important step toward identifying biomarkers that may ultimately be applied to monitor drug activity or therapeutic response in MASH.

## Methods

### Patient samples

Liver disease controls with significant steatosis (> 5%) on biopsy and healthy controls with abnormal liver biochemistry and/or elevated body mass index were excluded. Demographics and baseline characteristics of the study population are summarized in Table 1. For some focused analyses, we considered all 14 MASH subjects (Early (n = 8) or Late MASH (n = 6)) as one single group of Healthy controls (n = 4) patients, while for most of the validation study, Early Bariatric MASH (n = 8) and Late Bariatric MASH (n = 12) cohort were used as two unique groups or consolidated total MASH cohort. All studies were carried out in accordance with relevant guidelines and regulations and all the protocols were approved by the Institutional Review Board of the University of California San Diego. Informed consent was obtained from all participants and/or their legal guardians.

### Liver organoids and hepatocytes supernatants

Liver organoids (HemoSheare) and hepatocytes were provided by TAKEDA. We use the term ‘organoids’ to describe these 3D multicellular spheroid structures generated by co-culturing primary human hepatocytes, hepatic stellate cells, and macrophages. Unlike stem-cell–derived hepatic organoids, these spheroids do not self-renew or expand, but they provide a physiologically relevant multicellular environment to study liver-specific interactions and EV secretion. Human primary hepatocytes were cultured in optimal cell culture media following standard protocols. This included Williams medium with 21 mM glucose, dexamethasone, a penicillin–streptomycin cocktail, ITS + (a complex of insulin, transferrin, selenium, BSA, and linoleic acid), GlutaMAX™, and HEPES. For ‘obese’ hepatocyte/MASH induction (‘MASH-cocktail’), the media were the same as the healthy medium but with the addition of 300 µM oleic acid, 300 µM palmitic acid, and 1 ng/ml rhTNF-alpha. Liver organoids (HemoSheare) contain primary human hepatocytes, hepatic stellate cells and macrophages. Liver organoids supernatants were provided by TAKEDA (Induction media was used as per HemoSheare recommendations).

### Study assessments

Core liver biopsies were obtained at screening and were read by a single central reader (ZG). Histologic assessments included confirmation of the diagnosis, fibrosis staged according to a modified Ishak classification and the NASH Clinical Research Network (CRN) classification, and grading of steatosis, lobular inflammation, and hepatocellular ballooning according to the NAFLD Activity Score (NAS)^78^.

### Serum samples preparation

EVs were isolated following MISEV2018 guidelines using size-exclusion chromatography (SEC) as described previously^19,79^. SEC effectively reduces contamination from lipoproteins and protein aggregates. While minor non-EV proteins may still be present, subsequent analyses focused on proteins consistently enriched in EV fractions across all samples to minimize potential confounding effects on proteomic profiling. Briefly, 500ul of crude serum sample were centrifuged at 400*g* for 10 min at 4℃. Supernatants were transferred into new tubes and pellets discarded. Supernatants were further centrifuged at 2500×*g* for 15 min, followed by 12,000×*g* for 30 min at 4℃. Supernatants were added to a qEVoriginal/35 nm SEC column (iZON Science, Medford, MA, USA) for size-exclusion chromatography (SEC), according to the manufacturer’s instructions. Each sample group was assigned to a dedicated column. Samples were eluted through the column and fractions 1 to 6 (500 µL/fraction) were discarded. Fractions 7 to 9 (equal to 1.5 mL total volume), containing most of EVs, were collected and combined in one tube. Eluted EVs were concentrated six times in 3KDa Amicon filter units (cat. n. Z740186, Sigma-Aldrich, St. Louis, MO, USA) by centrifugation at 800 × g for 25 min at 4 °C. The resulting protein- and lipoprotein-free concentrated samples were used for Western Blotting, NanoView chip, NanoFCM, ONI high resolution microscopy. The supernatants from organoids or hepatocytes cultures will be processed the same way for the SomaScan array or Western blotting analysis. However, to measure and characterize the EVs from supernatants via ExoView® the 3KDa Amicon filter units (cat. n. Z740186, Sigma-Aldrich, St. Louis, MO, USA) were used and samples were concentrated by centrifugation at 800 × g for 10 min at 4 °C.

### Cryo-EM

For direct vesicle imaging, cryo-EM was performed. Lacey carbon EM grids were glow-discharged (30 s, 25 mA) in the Pelco EasiGlow machine to prepare samples for cryo-EM research. Using a Vitrobot Mark IV, an aliquot (3 L) of the sample’s aqueous solution was applied to the carbon side of the EM grid, which was then blotted for 2.0 s and plunge-frozen into the liquid ethane that had been precooled (FEI, USA). The samples are preserved in their natural condition and prevented from radiation harm by this method, which involves embedding them in a thin layer of amorphous ice.

The materials were examined using a cryo-electron microscope, the Titan Krios 60–300 TEM/STEM (FEI, USA), which is outfitted with a high-sensitivity TEM direct electron detector (DED) Falcon II (FEI, USA) and a Cs image corrector (CEOS, Germany). The EPU software (FEI, USA) was utilized in low-dose mode to reduce radiation damage during picture capture. Images were captured with the Falcon II DED at magnifications of 18,000 × and 37,000 × with defocus values of [-2 m; -5 m]. The maximum cumulative total dosage per picture was 50e/A2.

### Dynamic light scattering (DLS)

EV size was determined by measuring DLS with the Zetasizer system (Malvern Panalytical), and EV morphology was determined by TEM provided by the TEM facility at UCSD. Briefly, extracted EVs were diluted 10 × using DPBS and then transferred into a 346-well plate. After the laser and temperature equilibrium of the device (Dynapro Nanostar, WYATT Technology) was stabilized, we put 346- well plate into the DLS device to start measuring. For reproducibility and standardization, the parameter values were fixed as follows; Laser Wavelength (nm): 663.87, Temperature Controlled: yes, Peak Radius Low Cutoff(nm): 0.5, Peak Radius High Cutoff(nm): 5000, Auto-attenuation Time Limit(s): 60, Calculate D10/D50/D90: yes, Calculate Polydispersity: yes, Set temperature(C): 25, Wait (min): 5. The size or homogeneity of EVs was recognized through the radius or the % Intensity, respectively. The measurement with DLS was conducted with 10 acquisition of 5 s.

### Western blotting

Purified circulating EV proteins were extracted through five freeze/thaw cycles, which result in greater concentration of extracts and enrichment of low abundant proteins. Protein concentration was determined by micro-BCA protein assay kit (catalog #23235; Thermo Scientific, Waltham, MA), according to the manufacturer’s instructions. Further information can be found in the approximatively 10 µg of EV protein lysates were solubilized in Laemli buffer, resolved by a 4–20% Criterion Tris–HCl gel electrophoresis system (Biorad, Hercules, CA, USA) and transferred to a 0.2 µm nitrocellulose membrane (Biorad, Hercules, CA, USA). Primary TSG101 and CD63 (Genetex, 1∶200) were incubated overnight at 4 °C. Proteins were visualized by Li-Cor system via Image Studio Light v_5.2.

### NanoView chip assays

Organoid culture supernatants were run on the affinity chips as per manufacturer’s protocol. Briefly, detection of the Single Particle Interferometric Imaging Sensor (SP-IRIS) which allows multiplexed phenotyping and digital counting of various populations of individual EVs (> 50 nm) were captured on a microarray-based solid phase chip. The standard array chips were used for this measurement. Organoid culture media were incubated overnight at RT on the sensor chips. The unbound particles were then removed by series of washes including PBS-T and three times PBS. Each wash was done on the shaker for three minutes. After washing, chips were incubated with the specific fluorescently labeled using BSA in PBS-T. Following 1.5 h incubation time the washes removed unbound fluorescently labeled antibody once in PBS-T and three times PBS and Finally in Millipore water. The chips were then dried. Chips were imaged using ExoView® reader (NanoView Biosciences). Analysis was performed using NanoView analysis software (ExoView® Version 3.0 Software Suite).

By subtracting the signals observed after and before sample incubation, the effective vesicle binding to the capture antibodies was determined. Three spot duplicates’ net values were averaged. ExoView Reader was also used to detect EV immunostaining. The ExoView analyzer overlays images from the respective fluorescence channels providing quantitative information on biomarker colocalization on each individual vesicle. To colocalize up to four markers on a single EV, the program combines information from the respective fluorescence channels with the knowledge about which antibody spot the vesicle is bound to, in order to colocalize up to 4 markers on a single EV.

The protein of interest was measured alongside the tetraspanin markers. Co-expression of both markers suggest that the particle is derived from EVs. Exosomes that have been captured on ExoView® chip were fixed, permeabilized and probed for cargo protein staining. This allows the antibodies that detect cargo to be used. The presence of the cargo proteins can be colocalized on individual exosomes alongside additional surface markers. Finally, the chips were imaged with the ExoView R100 reader using ExoViewer 2.5.5 acquisition software, and the data were analyzed using ExoViewer 2.5.0 with sizing thresholds set to 50 to 200 nm diameter. Normalized particles were counted as the differences between post-scan and pre-scan. The pre-scan value represents the number of fluorescently labeled antibodies without any extracted samples.

### nanoFCM and flow cytometry

We adhered to MISEV guidelines when analyzing vesicles by flow cytometry^18^. In nanoFCM, cytometer excitation and emission light paths were aligned using 100-nm silica nanoparticles immediately before analyzing vesicle samples. A 488-nm laser diode was used to illuminate the sample; side-scattered light and GFP fluorescence were collected in separate channels using avalanche photodiode detectors. Distilled water was used as sheath fluid. We empirically determined acquisition parameters (laser power, detection threshold, detector gain) to minimize background using buffer-only controls to yield a background event rate of 2–4 events per second. Sample injection pressure was maintained at 1.5 kPa, yielding well-separated particle detection events. All the samples were analyzed using identical parameters on the same day by nanoflow software analyzer or FlowJo_v10.8.0. Each sample was serially diluted yielding 50–100 detection events per second.

### Super-resolution microscopy (ONI)

The Nanoimager (Oxford Nanoimaging) was calibrated for dSTORM using 100 nm Tetraspek microspheres (Invitrogen T7279) diluted in water. Calibration beads were viewed under the Nanoimager using a 405/473/561/640 nm laser configuration with a 100X oil‐objective lens. X, Y, and Z axes errors were obtained after the 3‐D mapping calibration was completed. The software analysis program used was the Nanoimager Software v 1.4.8740 (Oxford Nanoimaging). Channels were pseudocolored (Channel 0 = light blue #55aaff; Channel 1 = bright red #ff0011) for RG‐colorblind individuals. The staining of the samples by the video was recorded using ONI software and then converted into an MPEG4 or WMV file using HandBrake v 1.3.3. The pictures were stitched for better visualization of the output.

### SOMAscan array

The multiplex aptamer-based protein array SOMAscan test, with 7,288 SOMAmer reagents that has over 300 control that leaves 6,596 unique analytes. All samples passed the established technical quality control according to the standard quality control protocols at SomaLogic. Raw data were normalized by using the SomaLogic methods^80,81^. Briefly, normalization and calibration are routine numerical procedures developed to remove systemic biases in the raw assay data. Further normalization was applied: hybridization control and Intraplate median signal.

### Modelling

The machine learning model was based on input data that included the initial discovery included 4 healthy and 13 MASH patients’ datasets. Since the dataset was imbalanced, we implemented synthetically generated data into the model to oversample the number of healthy with an additional 13 synthetic dataset. The top 19 proteins were selected as an input from early and late MASH subjects versus healthy controls. Four subjects without histologically proven MASH and 13 with MASH were correctly classified as absence or presence of the disease respectively. In addition, our algorithm effectively classified all 13 synthetic datasets, showcasing its robustness. To ascertain convergence, we introduced a loss function projection, and notably, convergence was observed after 2.5 k Epochs. To test the model, we feed the data of known patient profile to assess probabilities for each class. With the help of the synthetic data generation tools, we increased the total number of the testing set to 37 (MASH n = 18 and synthetic healthy data n = 19). The neural network (NN) analysis for predicting the presence or absence of MASH successfully identified all MASH patients as class 1. Classification report can be found in supplementary material. Briefly, in developing the classification neural network, we adhered to standard practices in machine learning. The output layer is structured as a one-hot encoding, with neurons corresponding to the number of classes. ReLU activation functions were employed for the hidden layers, while Softmax activation was used exclusively for the output layer. Categorical cross-entropy served as the cost function, and the ADAM optimizer was selected for model optimization. The model, developed using Python and Keras integrated with TensorFlow, showcased a robust architecture with 19 input biomarkers, 7 neurons in the hidden layer, and 2 neurons in the output layer. Training involved 20,000 epochs, resulting in a converging loss function. The confusion matrix provided additional insights into the model’s performance and overall accuracy.

### Statistical analysis

Experimental replicates, including the number of EV isolations used, are defined in the figure legends for each experiment. Numbers of samples or pulled samples analyzed for specific experiments are reported in the figure legends as well. While formal statistical assessment of normality is limited for experiments with small sample sizes, the distributions appeared approximately symmetrical. Statistical analyses were conducted using Python or GraphPad Prism 7 v_9.3.0. Differences between two groups were compared by a two-tailed Student’s t-test if data had a normal distribution or a Mann–Whitney test if the data deviated from the normal distribution. Values are expressed as mean ± standard deviation (SD) or mean ± standard error of the mean (SEM), as specified in the figure legends. Box and whisker plots span the maximum and minimum values, with the box showing 25th–75th percentiles. The bar marks the median value. Differences with a p < 0.05 were considered significant. * represents significance (*p* < 0.05), **p < 0.01, ***p < 0.001, ****p < 0.0001).

## Supplementary Information

Supplementary Information 1.

Supplementary Information 2.

## Data Availability

The primary data supporting the findings of this study, are provided within the article and its Supplementary Information. All additional data generated during the study, including the source data for the figures, are publicly available in the Zenodo repository under accession number **10.5281/zenodo.18009920** . All additional information is available from the corresponding author upon reasonable request *.*

## Code availability

The custom code used in this study is available on GitHub: https://github.com/aleksanarepo/Protein-Based-Disease-Classification-Model
