# Supplementary material for: Profiling of extracellular vesicles from primary hepatocytes, organoids, and mash patients identifies cell injury-specific signatures
Source: Sci Rep. 2026 Jun 3;16:17147. doi: 10.1038/s41598-026-40490-x (PMC13233857; doi:10.1038/s41598-026-40490-x)
Supplement: Supplementary file 2 — Supplementary Information 2. [file 41598_2026_40490_MOESM2_ESM.docx]

Profiling of Extracellular Vesicles from Primary Hepatocytes, Organoids, and MASH Patients Identifies Cell Injury-Specific Signatures.

**Authors:** Aleksandra Leszczynska^1^, Benedikt Kaufmann^1^, Hana Sung^1^, Christian Stoess^1^, Agustina Reca^1^, Andrea Kim^1^, Yeon-Kyung Choi^1^, Chelsea Tran^1^, Sung-Eun Kim^1^, Davide Povero^2^, Bruce Wolfe^3^, Trevor Crafts^3^, Akiko Eguchi^4,5^, Ariel E. Feldstein^1,6^

^1^ Department of Pediatrics, University of California, San Diego, USA.

^2^ Division of Gastroenterology and Hepatology, Mayo Clinic, Rochester, MN, USA

^3^ Department of Surgery, Oregon Health and Science University, Portland

^4^ Department of Gastroenterology and Hepatology, School of Medicine, Mie University, Mie, Japan; akieguchi@med.mie-u.ac.jp

^5^ Biobank Center, Mie University Hospital, Mie, Japan; akieguchi@med.mie-u.ac.jp

^6^ Pfizer, Internal Medicine Research Unit, 1 Portland St, Cambridge, MA 02139

**Supplementary methods and figures**

**
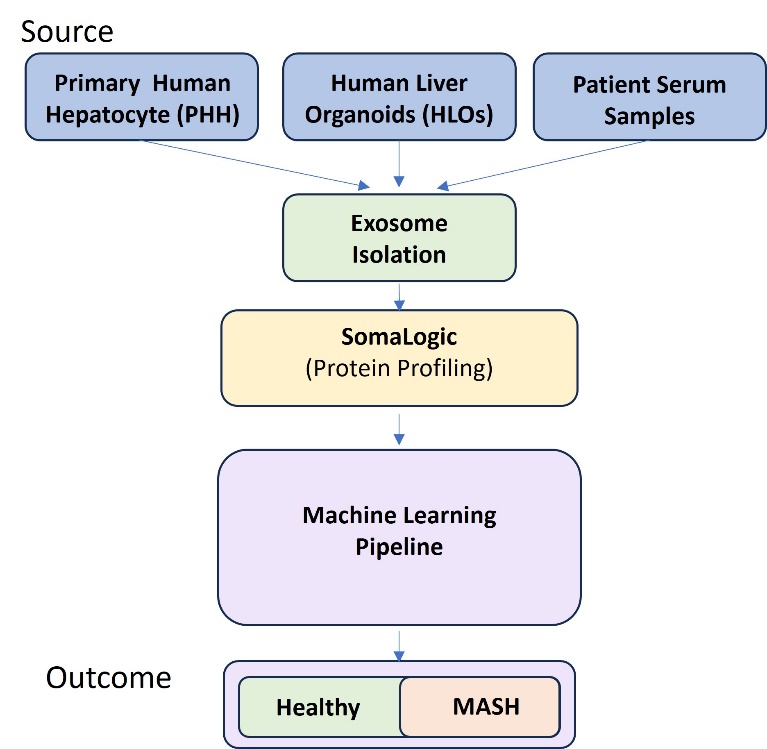
**

**Simplified schematic of the study workflow.**

Overview of the experimental and analytical pipeline: extracellular vesicles (EVs) were isolated and profiled from primary human hepatocytes (PHH) supernatants, human liver organoids (HLO) supernatants and serum from patients with MASLD/MASH. EV protein cargo was characterized and integrated across models to identify biologically relevant signatures. Machine learning models were then applied to evaluate diagnostic potential of the identified biomarkers.

- 1. **Feature model selection**

Proteins that were differentially expressed successfully separated MASH from healthy controls, demonstrating a clear distinction in the EV proteome (Fig 3 & 2 in the main body of the manuscript). Notably, these dysregulated proteins exhibited distinct trends throughout the disease spectrum. We leveraged the Ridge regression algorithm to manage multicollinearity and improve model accuracy, while incorporating feature selection methods to identify predictors^1^. Using frequency-based selection in circulating EVs, and tuning lambda parameter we confirmed feature coefficients of the 19 proteins - ‘HP', 'HPX', 'SERPINA7', 'SERPINA3', 'GSTA2', 'PTPA', 'FABPA', 'CLC1B', 'FRIH' (FTH1), 'ARG1', 'IGFBP', 'TIMP2', 'FYN', 'IL27RA', 'PPBP', ’OSMR’, 'S100A4', 'SAA1', 'ANKRD (Supplementary Fig. 1a). Furthermore, we identified overlapping key features from EVs isolated from both organoids and hepatocytes (Supplementary Fig. 1b). These features include SERPINA3, PPBP, ANKRD, IGF1, FABP4, FTH1, S100A4, PTPA, and FYN. This overlap not only boosts the predictive power of our model but also provides compelling evidence suggesting that these markers could originate from liver circulating EVs. Subsequently, markers from Ridge regression approach were used to build a Neural Network model for Healthy control vs MASH. Meanwhile, the k-Nearest Neighbors (k-NN) model or Logistic Regression models played a pivotal supportive role in comparing the performance and accuracy of the Neural Network. (model provided in supplementary materials 1.3 and 1.2 respectively).


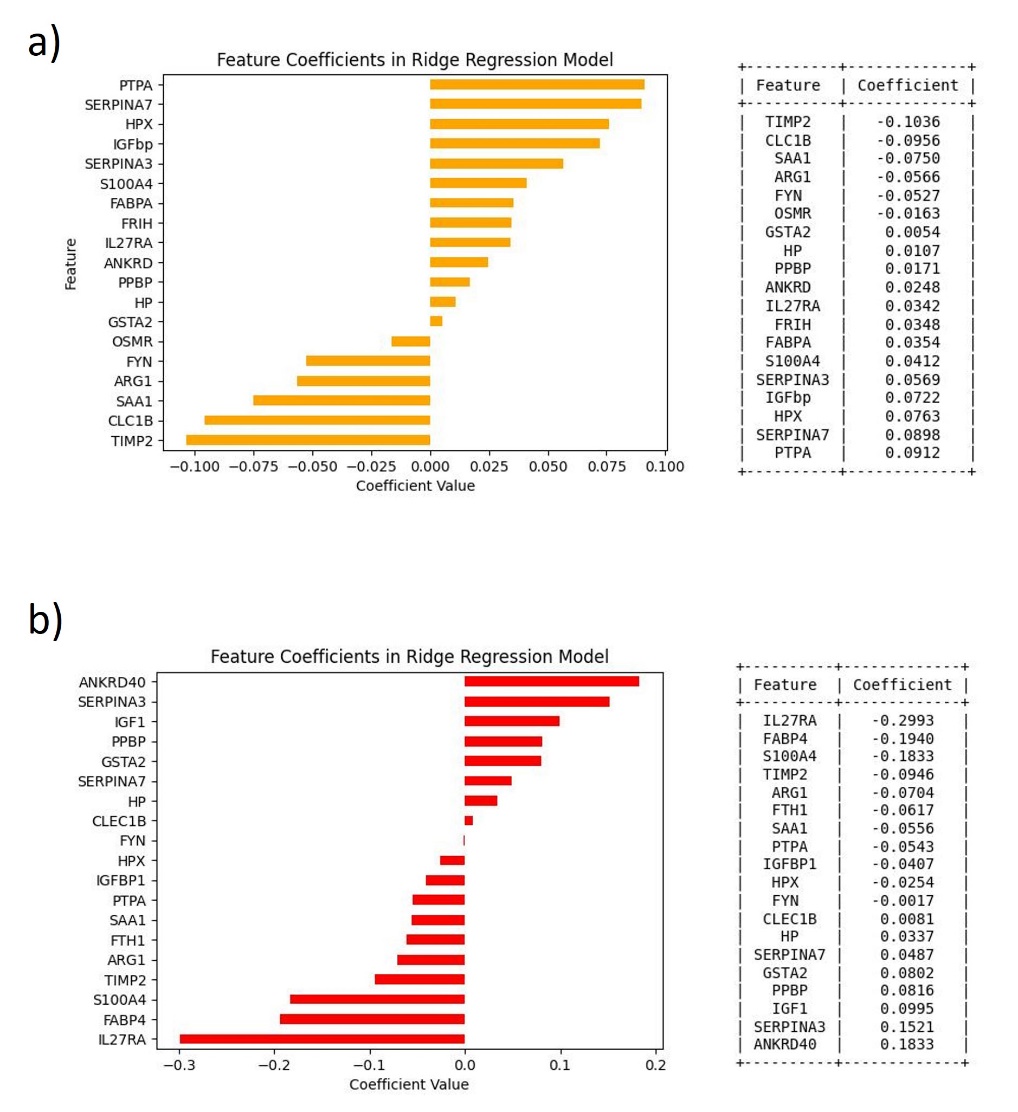


**Supplementary Figure 1. The Ridge regression algorithm's performance was evaluated on input features derived from two distinct data sources: a) Circulating EVs: The selected data from circulating EVs were analyzed to determine the efficacy of the feature selection process in distinguishing between different conditions. b) Primary Human Hepatocyte PHH and HLO EVs: The selected data from PHH and HLO EVs were assessed by the Ridge regression algorithm's performance across untreated and MASH treated conditions.**

**1.2** **Deep Learning Model Accurately Predicts MASH Patient Outcomes: Validating Proteomic Data from PHH, HLO, and Circulating EVs for Reproducible Diagnostic Signatures.**

In developing the classification neural network, we adhered to standard practices in machine learning. The model was trained and tested on different datasets. With NNs, there are no guidelines to a minimum sample size, recommendations mostly being “the more the better”^2^. Model estimation was conducted using the training set, and testing was performed to validate the outcomes. The model’s output layer is structured as a one-hot encoding, with neurons corresponding to the number of classes. ReLU activation functions were employed for the hidden layers, while Softmax activation was used exclusively for the output layer. Categorical cross-entropy served as the cost function, and the ADAM optimizer was selected for model optimization.

The response variable, initially one-hot encoded, was prepared using Pandas' get_dummies function, converting the data frame into a NumPy array. Class balancing was achieved by using an augmented dataset. During this process, all available data from the first MASH cohort was utilized for training.

Upon loading the data and encoding the response variable, we inspected the one-hot representation to ensure correctness, particularly verifying that the second neuron in the output layer denoted the presence of a disease ([0,1]) or its absence ([1,0]).

The neural network architecture was designed with 19 input biomarkers, 7 neurons in the hidden layer, and 2 neurons in the output layer. Python, leveraging Keras (version 2.14.0) integrated with TensorFlow, facilitated model development within the Google cloud environment. The Sequential model was instantiated, followed by the addition of layers, specifying the activation functions, and model compilation with categorical cross-entropy loss and ADAM optimizer.

The model summary revealed parameters for each layer, with 140 in the first dense layer, 16 in the second dense layer, and a total of 156 parameters.

Training the model involved 20,000 epochs on the provided training features and labels, with the loss function converging to zero, indicating successful model convergence. Subsequently, we initiated predictions and examined the weight and bias outputs. Further assessment of the model's performance involved evaluating its predictions against known outcomes in the Bariatric MASH cohort. To quantitatively evaluate the model's overall accuracy, a confusion matrix was constructed, revealing a 97% accuracy score in predicting test cases. This comprehensive evaluation solidifies the confidence in the neural network's ability to correctly classify disease and non-disease states in the given dataset.

In more detail, in the presented classification results, the model exhibits robust performance across both classes, providing a comprehensive evaluation of its predictive capabilities. The precision, recall, and F1-score metrics convey a balanced view of the model's effectiveness in classifying instances from each class.

Class 0 (Negative Class): *Precision:* 100%, *Recall:* 95%, *F1-Score:* 97%.

For the negative class, the model demonstrates impeccable precision, ensuring that all instances predicted as negative are indeed negative. Additionally, it captures a substantial portion (95%) of the actual negative instances, as indicated by the recall metric. The harmonized F1-Score reflects a robust balance between precision and recall for class 0.

Class 1 (Positive Class): *Precision:* 95%, *Recall:* 100%, *F1-Score:* 97%.

The positive class exhibits commendable precision, with 95% of instances predicted as positive being accurate. Furthermore, the model captures all actual positive instances, resulting in a perfect recall. The F1-Score underscores the harmonious equilibrium between precision and recall for class 1. This metric provides an assessment of the model's performance on the entire dataset.

- 1. **Performance Analysis of Logistic Regression on MASH training Cohort**

Candidate biomarkers were identified by Ridge selection, which were then used to generate the logistic regression model. For each observation in logistic regression, the model calculates the probability of the observation belonging to one of the two classes (0=HC, 1=MASH) using the logistic function. The predicted class is determined by applying a threshold (0.5) to this probability. The performance of the logistic regression classifier is dependent upon the number and quality of the predictor variables used to construct and train the model. In this study, 2 to 5 variables are selected (Supplementary Fig. 2) for each model to estimate the parameters that maximize the likelihood of the observed outcomes. As per our observations, we can confidently state that regardless of the variables chosen for this model, it provided high confidence in predicting negative or positive power output. The area under the receiver operating characteristic curve (AUROC) and the 95% confidence intervals (CI) were consistently high, indicating robust model performance.


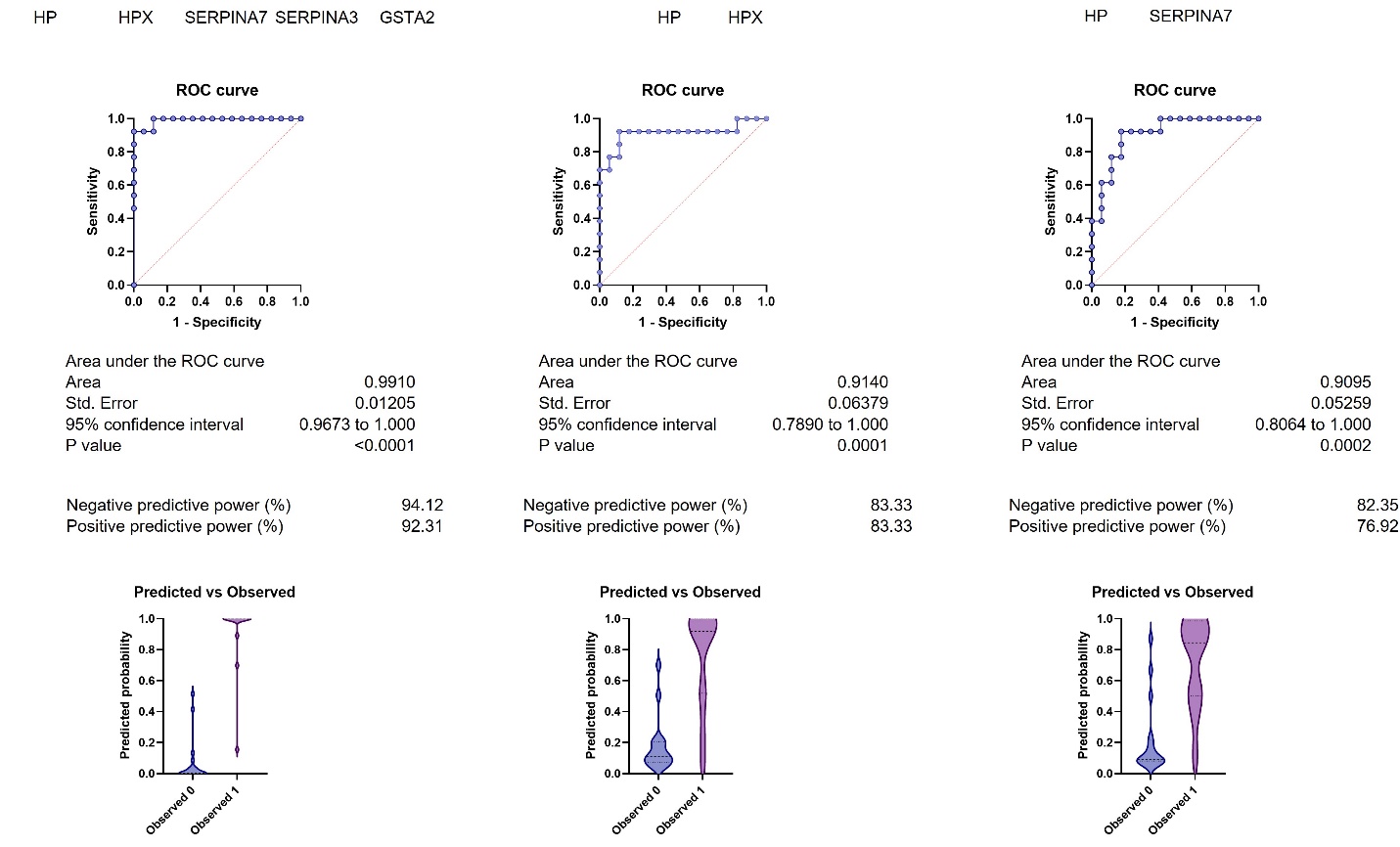

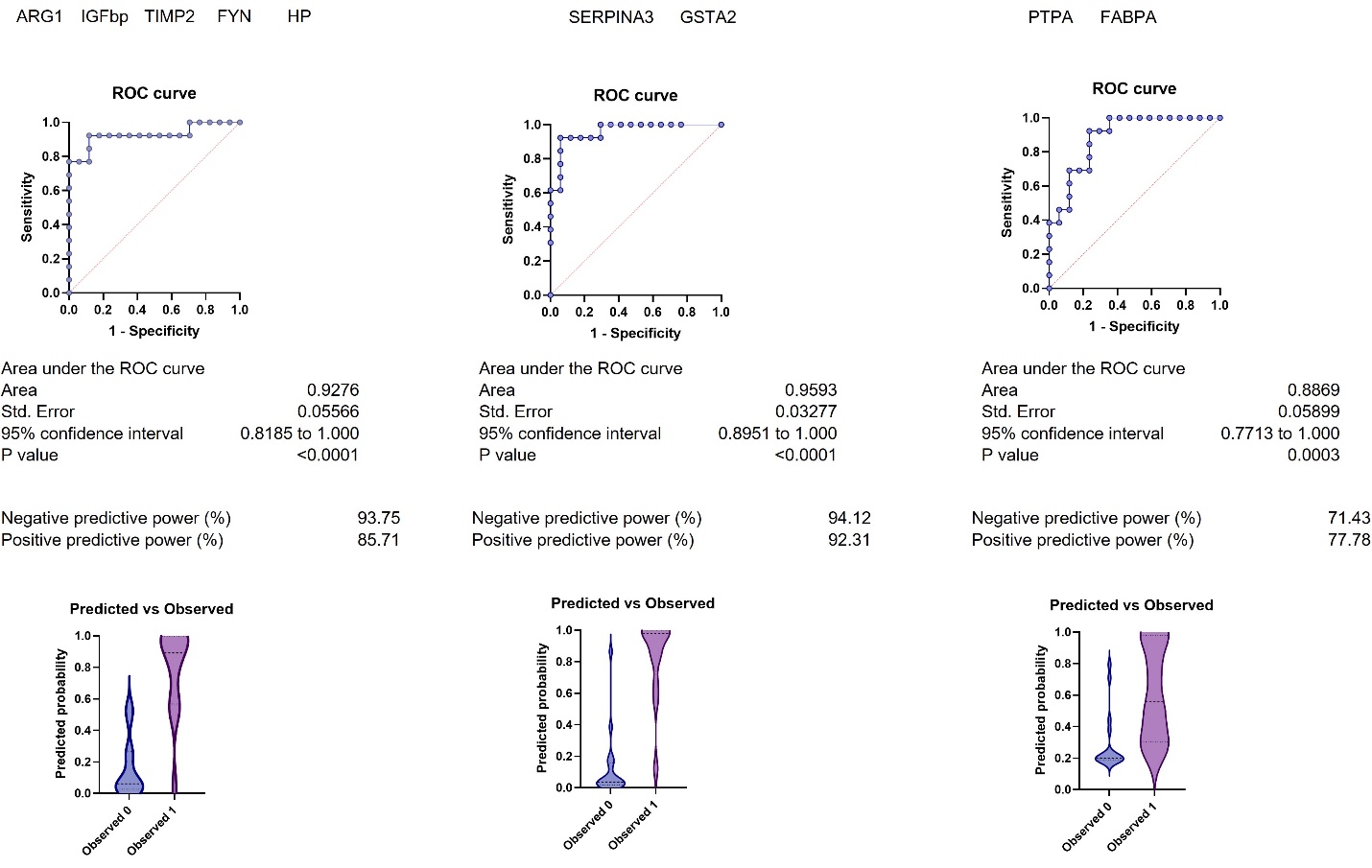

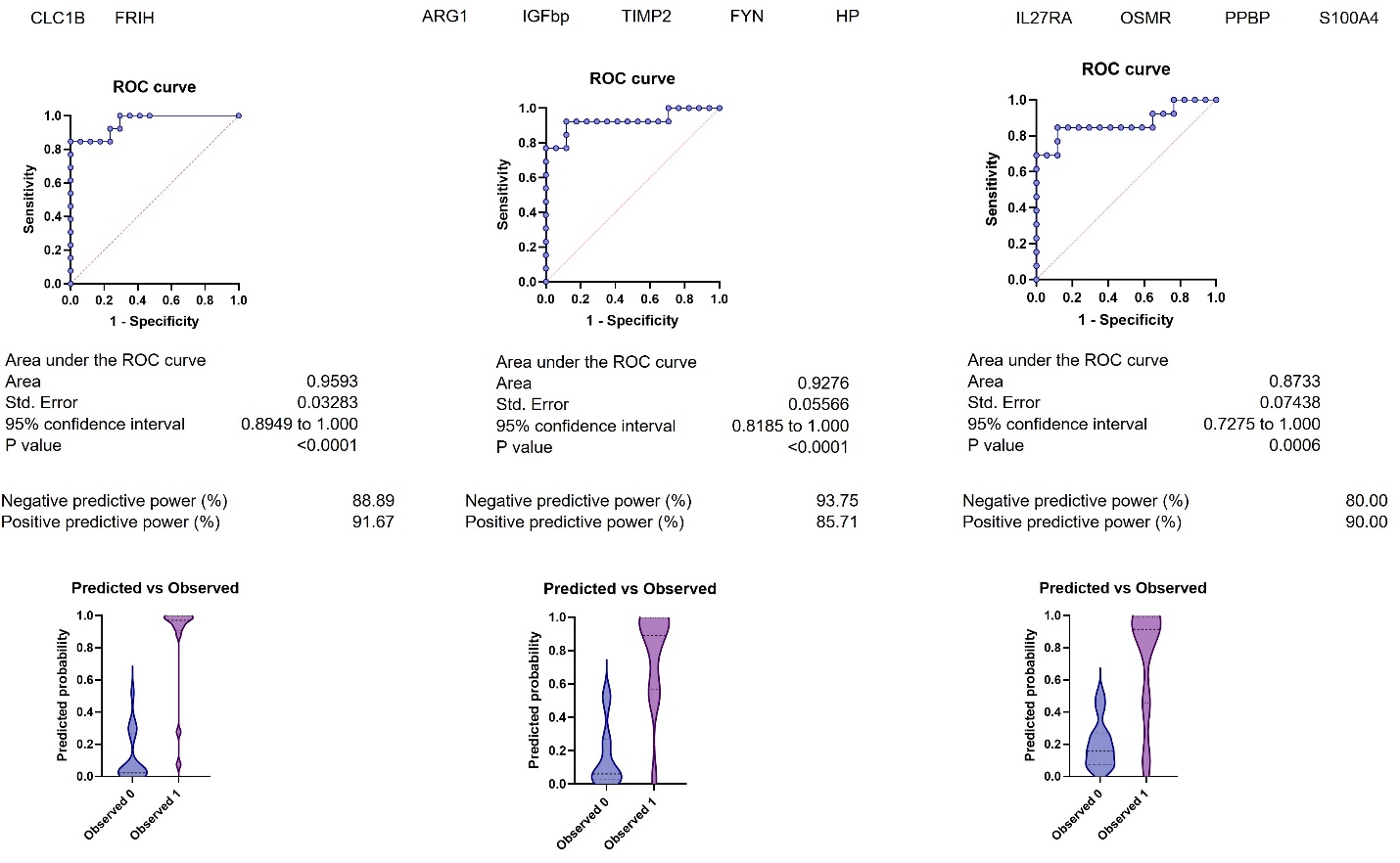


**Supplementary Figure 2. Validation metrics of the Logistic Regression model are illustrated as follows:**

**a) ROC Curves: The graphs show the Receiver Operating Characteristic (ROC) curves and predictive performance for the selected sets of variables.**

**b) Violin Plots: The plots present the distribution of predicted probabilities for observed outcomes (observed 0 = HC, observed 1 = MASH) for each biomarker.**

**1.4 Performance Analysis of k-NN Classification on Bariatric Surgery Cohort**

We used the k-Nearest Neighbors (k-NN) method as a benchmark to compare the performance of neural networks. Given the high dimensionality of the feature space, we selected a set of suitable features from a panel of 19 biomarkers for k-NN prediction. To further elucidate the relationships between features and outcomes, we constructed a correlation matrix. This analysis served as the basis for developing a binary classification model. The correlation matrix is visualized in a heatmap, which depicts the association between attributes and class variables (Supplementary Fig. 3a). Notably, a correlation value of 1 signifies a patient diagnosis of MASH. Next, we decided to closely examine two predicted variables: HP and SERPINA7. This also allowed us to better visualize their associated decision boundaries (Supplementary Fig. 3b). We selected k=7 for our model, as it resulted in the lowest error rate and helped minimize the risk of overfitting or underfitting. Additionally, the weight-by-distance method showed a slight improvement in performance. We split the data into training (2/3) and testing (1/3) sets to apply the k-NN algorithm to the test set. This enabled us to classify whether a patient has MASH. To enhance visualization, we applied principal component analysis (PCA) to all selected features, displaying the decision boundaries in the PCA1 and PCA2 plane. This provided clearer insights into the model's behavior and performance (Supplementary Fig. 3b). A pairplot of all features is included as a PDF file, where a value of 1 indicates the presence of MASH.


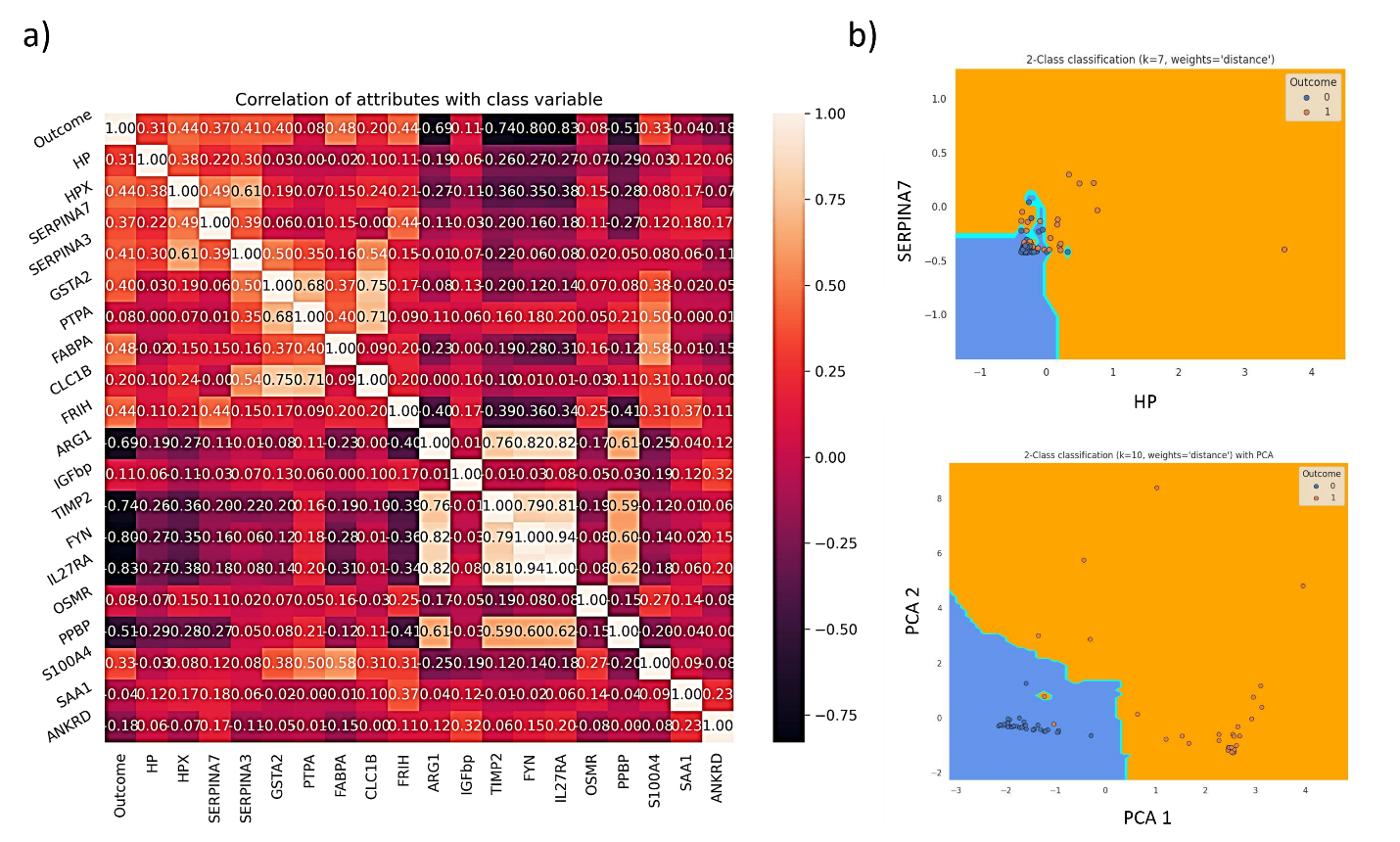


**Supplementary Figure 3. Validation metrics for the k-Nearest Neighbors (k-NN) model include the following:**

**a) A heatmap illustrating the correlation between attributes and the class variable, which provides insight into the relationships between features and their influence on classification outcomes.**

**b) The class decision boundaries and performance metrics of the testing data were assessed by applying the k-NN algorithm. This analysis compared results using two variables or Principal Component Analysis (PCA) of all features to evaluate the impact of dimensionality reduction on model performance.**

The underlying variance is illustrated through the communality and covariance structure of the observable data, resulting in the identification of four factors. This suggests the presence of potentially fewer unobservable variables, which are our common factors. Using Kaiser’s Criterion/Eigenvalues test, we assessed the suitability of the data for structure detection. This test highlights the proportion of variance in the variables that may be attributed to underlying factors. Specifically, cell stress influences the following variables: PTPA, GSTA2, and S100A4 (Supplementary Fig. 4).

**
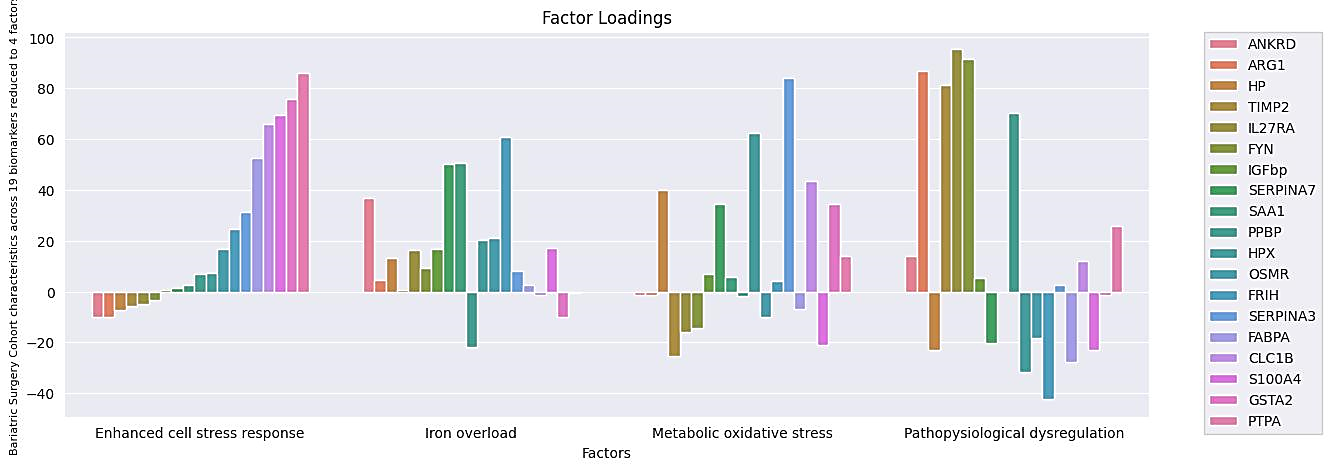
**

**Supplementary Figure 4. KMO Factor Analysis Reveals Four Key Factors in Bariatric Surgery Data, Highlighting Cell Stress Influence the following examples of the variables: PTPA, GSTA2, S100A4, CLEC1B, and FABP4.**

**
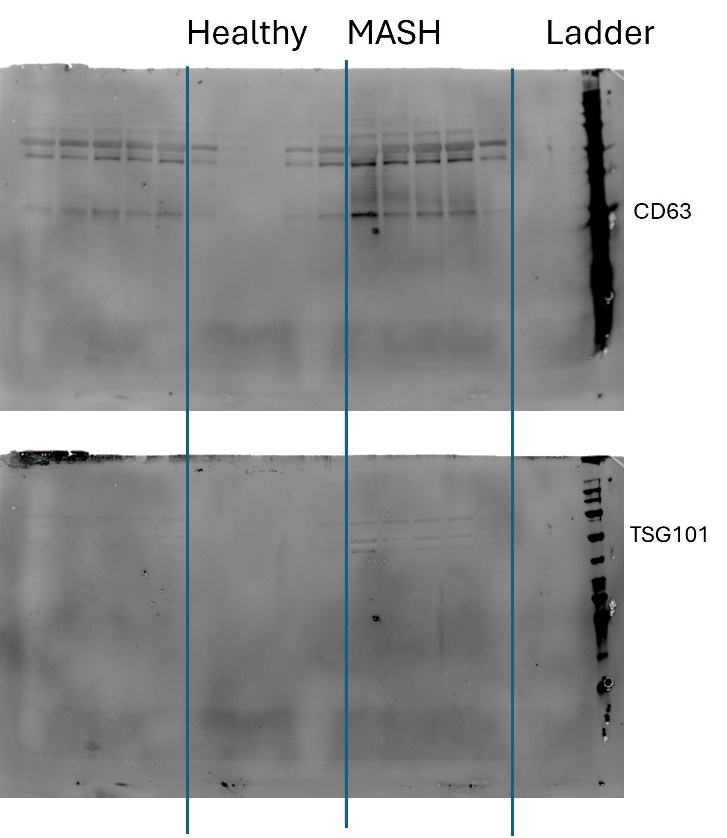
**

**Supplementary Figure 5. Western blot analysis of EV markers shows CD63 and TSg101 in Healthy control and MASH derived circulating EV protein lysates.**

**Reference**

1 Eliot, M., Ferguson, J., Reilly, M. P. & Foulkes, A. S. Ridge regression for longitudinal biomarker data. *Int J Biostat* **7**, Article 37, doi:10.2202/1557-4679.1353 (2011).

2 Koutsoukas, A., Monaghan, K. J., Li, X. & Huan, J. Deep-learning: investigating deep neural networks hyper-parameters and comparison of performance to shallow methods for modeling bioactivity data. *J Cheminform* **9**, 42, doi:10.1186/s13321-017-0226-y (2017).
